# Conversational Agent Interventions for Mental Health Problems: Systematic Review and Meta-analysis of Randomized Controlled Trials

**DOI:** 10.2196/43862

**Published:** 2023-04-28

**Authors:** Yuhao He, Li Yang, Chunlian Qian, Tong Li, Zhengyuan Su, Qiang Zhang, Xiangqing Hou

**Affiliations:** 1 Institute of Applied Psychology, College of Education Tianjin University Tianjin China; 2 Laboratory of Suicidology Tianjin Municipal Education Commission Tianjin China; 3 Shenzhen School, Sun Yat-sen University Shenzhen China

**Keywords:** chatbot and conversational agent, mental health, meta-analysis, depression, anxiety, quality of life, stress, mobile health, mHealth, digital medicine, meta-regression, mobile phone

## Abstract

**Background:**

Mental health problems are a crucial global public health concern. Owing to their cost-effectiveness and accessibility, conversational agent interventions (CAIs) are promising in the field of mental health care.

**Objective:**

This study aims to present a thorough summary of the traits of CAIs available for a range of mental health problems, find evidence of efficacy, and analyze the statistically significant moderators of efficacy via a meta-analysis of randomized controlled trial.

**Methods:**

Web-based databases (Embase, MEDLINE, PsycINFO, CINAHL, Web of Science, and Cochrane) were systematically searched dated from the establishment of the database to October 30, 2021, and updated to May 1, 2022. Randomized controlled trials comparing CAIs with any other type of control condition in improving depressive symptoms, generalized anxiety symptoms, specific anxiety symptoms, quality of life or well-being, general distress, stress, mental disorder symptoms, psychosomatic disease symptoms, and positive and negative affect were considered eligible. This study followed the PRISMA (Preferred Reporting Items for Systematic Reviews and Meta-Analyses) guidelines. Data were extracted by 2 independent reviewers, checked by a third reviewer, and pooled using both random effect models and fixed effects models. Hedges *g* was chosen as the effect size.

**Results:**

Of the 6900 identified records, a total of 32 studies were included, involving 6089 participants. CAIs showed statistically significant short-term effects compared with control conditions in improving depressive symptoms (*g*=0.29, 95% CI 0.20-0.38), generalized anxiety symptoms (*g*=0.29, 95% CI 0.21-0.36), specific anxiety symptoms (*g*=0.47, 95% CI 0.07-0.86), quality of life or well-being (*g*=0.27, 95% CI 0.16-0.39), general distress (*g*=0.33, 95% CI 0.20-0.45), stress (*g*=0.24, 95% CI 0.08-0.41), mental disorder symptoms (*g*=0.36, 95% CI 0.17-0.54), psychosomatic disease symptoms (*g*=0.62, 95% CI 0.14-1.11), and negative affect (*g*=0.28, 95% CI 0.05-0.51). However, the long-term effects of CAIs for the most mental health outcomes were not statistically significant (*g*=−0.04 to 0.39). Personalization and empathic response were 2 critical facilitators of efficacy. The longer duration of interaction with conversational agents was associated with the larger pooled effect sizes.

**Conclusions:**

The findings show that CAIs are research-proven interventions that ought to be implemented more widely in mental health care. CAIs are effective and easily acceptable for those with mental health problems. The clinical application of this novel digital technology will conserve human health resources and optimize the allocation of mental health services.

**Trial Registration:**

PROSPERO CRD42022350130; https://tinyurl.com/mvhk6w9p

## Introduction

### Background

To promote mental health for everyone, everywhere, the World Health Organization’s most recent global mental health report strives to motivate and guide revolutionary action [1,2]. According to estimates by the World Health Organization, in the first year of the COVID-19 pandemic, the incidence of both depression and anxiety disorders increased by more than 25% [3]. However, mental health services are few and still underutilized globally [4-6]. This was because traditional face-to-face mental health care still had many limitations, such as expensive treatment, lack of experienced therapists, poor service quality, geographical constraints, and delayed treatment [7,8], and the resulting stigma and discrimination are also considered the most important barriers to providing mental health services [9,10].

Because of their increased acceptance and accessibility, digital mental health interventions have emerged as an important research area with evidence-based psychotherapies implemented on digital platforms [11]. Conversational agent interventions (CAIs) [12] were the new wave of digital mental health interventions to cope with the insufficient and inadequate mental health services [1,13]. Substantial human health resources will be saved if the CAIs are proven effective and suitable for widespread implementation [14].

Software programs known as conversational agents (CAs) use artificial intelligence techniques to simulate human behavior and offer a task-oriented framework with evolving dialogue able to engage in conversation [15]. CAs are equipped with computer models that range from succinct decision trees, where different responses to a questionnaire result in different responses from the CAs to machine learning–based and natural language processing–based algorithms that classify real-time multimodal input into a user’s emotional state, allowing the CAs to respond empathically [16,17]. One of the most crucial features of CAs in mental health was interactivity, which was designed to promote a conversational process instead of a single psychological education, in which inputs and outputs are generated in unrestricted natural language rather than predefined or preprogrammed choices or messages [12]. The other was automation, which means that most CAs for mental health issues can independently provide automated services to users without the participation and guidance of human [18].

Certain studies claimed that CAIs can help users feel accompanied and understood [19,20]; several studies found that users establish therapeutic bonds with chatbots [21-24]; and other studies revealed some hazards associated with CAIs, including *misunderstanding* that may result in inefficient or even harmful interventions, a lack of crisis warning systems, and a lack of privacy protection [25]. An increasing number of CAIs have emerged in recent years, as the digital medical and mobile health fields have grown [26], solving and improving a larger range of mental health issues in addition to depression and anxiety. Therefore, a thorough systematic review and meta-analysis of CAIs for mental health problems are urgently needed.

### Objectives

In this study, we outlined the clinical and nonclinical features of CAIs in mental health using a systematic review. We then evaluated the short- and long-term efficacy of CAIs for different mental health outcomes via a meta-analysis and assessed whether various characteristics related to the intervention and sample moderated the observed effect sizes.

## Methods

### Study Protocol and Registration

We have reported the findings in accordance with the PRISMA (Preferred Reporting Items for Systematic Reviews and Meta-Analyses; Table S1 in Multimedia Appendix 1 [27-58]) guidelines [59]. The study protocol was registered in the PROSPERO database (CRD42022350130).

### Search Strategy

As mentioned in the initial registration, we searched 6 major web-based databases (Embase, MEDLINE, PsycINFO, CINAHL, Web of Science, and Cochrane), dated from the establishment of the database to October 30, 2021, and updated to May 1, 2022, using the following search terms: (“conversational agent*” OR “conversational system*” OR “dialog system*” OR “dialogue system*” OR “assistance technolog*” OR “relational agent*” OR chatbot* OR “automat*” OR “virtual human*” OR “virtual agent” OR “virtual coach” OR “virtual therap*” OR avatar OR “artificial Intelligence”) AND (“depress*” OR “anxiety” OR “agoraphobia” OR “phobia*” OR “panic” OR “mental health” OR “mental illness*” OR “mental disorder” OR psycholog* OR “affective disorder*” OR “bipolar” OR “mood disorder*” OR “psychosis” OR “psychotic” OR “schizophre*” OR “well-being” OR “well-being” OR “quality of life” OR “self-harm” or “self-injury” OR “stress*” OR “distress*” OR “mood” OR “loneliness” OR “social isolation” OR “autism” OR “suicid*” OR “cogniti*” OR insomnia OR emotion* OR affect*) AND (“randomized trial*” OR “controlled trial*” OR “randomised trial*” OR RCT OR RCTs). In addition, the reference lists of the included original studies and previous reviews were manually searched to identify any further eligible studies. To reduce the possibility of publication bias, eligible published and unpublished papers were searched for inclusion.

### Eligibility Criteria

#### Participants

Participants were categorized as (1) clinical sample (from outpatients or inpatients in psychiatric hospitals), (2) symptomatic sample (from those at a diagnostic or subthreshold level of mental health problems), and (3) general sample (from universities, companies, or communities with a wider range of recruitment scales and standards). People with physical illnesses who did not have mental health problems were excluded.

#### Interventions

Interventions with four types of CAs were eligible: (1) chatbot, a software program that simulates conversations with users through text or voice depending on artificial intelligence [60], such as ELIZA (Massachusetts Institute of Technology), the first chatbot, via which users can input text to simulate a conversation with a Rogerian psychotherapist [61]; (2) embodied CA (ECA), the digital characters that simulate key properties of human face-to-face conversation, both verbal and nonverbal (eg, speech, gestures, and facial expressions) [62], called virtual human [63] and digital human [64]; (3) CA in virtual reality (VR), which offers a safe, convenient, and accessible medium to the controlled exposure to anxiety-inducing stimuli within a VR environment to deliver exposure-based behavioral treatments [65]; and (4) avatar, referring to the CA in avatar therapy, a new wave of relational approaches, now used mainly to treat auditory verbal hallucinations and depressive symptoms, allowing patients to interact with a digital representation (avatar) whose speech closely matches the pitch and tone of the persecutory voice and gradually gains increased power and control within the relationship [27].

#### Comparators

Control conditions were categorized as (1) active controls (therapist-led interventions, other CAIs, or treatment as usual), (2) information or attentional controls (self-help e-book or text), and (3) passive controls (waitlist [WL] or assessment only).

#### Outcomes

Eligible studies were those that reported at least 1 mental health outcome and provided the outcome data required to calculate the effect size. Studies were excluded if they focused on physical illness or physical health. We selected and analyzed the following mental health outcomes: depressive symptoms, generalized anxiety symptoms, specific anxiety symptoms (phobia symptoms, social anxiety symptoms, and panic symptoms), quality of life or well-being, general distress, stress, mental disorder symptoms (substance use disorder symptoms, attention-deficit/hyperactivity disorder symptoms, and psychotic symptoms), psychosomatic disease symptoms (chronic pain symptoms and irritable bowel syndrome symptoms), and positive and negative affect.

#### Study Design

The eligible studies were randomized controlled trials (RCTs). Studies were excluded if they met the following criteria: (1) aimed at assessment, management, medical skill training, or health knowledge access and (2) only focused on technology, engagement, usability, or user experience.

### Data Extracting

The following data were extracted: (1) basic study characteristics (including the first author, publication date, country, and mental health problems), (2) participant characteristics (including target sample, sample size, age, and gender), (3) intervention characteristics, (4) outcome measures, and (5) engagement and user experience.

Study results related to mental health outcomes were extracted in the form of means and SDs at baseline, postintervention, and follow-up when available, or, if not available, effect sizes. The study authors were contacted in case of missing or unclear information, and the study was excluded if they failed to provide the data.

It should be noted that in digital interventions, *interaction frequency* was usually used to represent dosage [66]. However, because different studies had different operational definitions of interaction frequency, to preliminarily explore the dose-response relationships of CAIs in mental health, we uniformly used the comparable data, average duration of interaction per day, to quantify the dosage in meta-regression.

### Quality Assessment

The quality of the trials was independently evaluated by 2 authors using the Cochrane Collaboration tool [67] to assess the risk of bias. The Cochrane Collaboration tool evaluates 7 domains (sequence generation, allocation concealment, blinding of participants and personnel, blinding of outcome assessment, incomplete outcome data, selective outcome reporting, and other risk of bias). For each domain, a rating of low (+), high (−), or unclear (?) was made for each trial. Only trials that received a low-risk rating on all 7 criteria were considered to have a low risk of bias. Trials were considered to have a high risk of bias if they were rated high in any bias domain other than performance bias, as blinding of participants and personnel is almost impossible in CAIs studies [68]. The risk of bias graph was drawn using Review Manager (version 5.4; The Cochrane Collaboration).

### Meta-analysis

Given the differences at baseline, the change-from-baseline scores were computed for each group to represent the pre-post efficacy. For each comparison between the treatment group and control group, the standardized mean difference was calculated, as there were different scales from the included study to measure the same outcome.

We chose Hedges *g* as the effect sizes [69]. A positive *g* indicates that the CAIs condition had better outcomes than the comparison condition. If means and SDs were not reported, the effect sizes were converted via other available statistics (eg, *d* or *η*^2^). If data from both intention-to-treat and completer analyses were presented, the former were extracted and analyzed. We analyzed the pooled effect sizes of outcomes at postintervention as the short-term efficacy and the pooled effect sizes of outcomes at follow-up as the long-term efficacy.

Stata (version 15; Stata Corp) was used to perform the analyses. Heterogeneity between studies was quantified by calculating the *I*^2^ statistic and Cochran *Q* statistic [70]. A random effects model was applied when substantial heterogeneity was observed (*P*<.05 or *I*^2^>50%); otherwise, a fixed effects model was used [71].

In a few studies, the same intervention condition was compared with >1 control condition (or vice versa), which may have artificially reduced the heterogeneity estimate and affected the pooled effect size, as these comparisons were not independent of each other. Thus, sensitivity analyses were run: only the comparison with the smallest effect size included in the meta-analysis or the largest effect size, which ensured that only 1 effect size per study was included in the analysis. We also performed an analysis only including low-risk articles to determine the pooled effect size after controlling for the risk of bias.

We conducted a subgroup analysis and meta-regression with the primary outcomes (depressive symptoms, generalized anxiety symptoms, specific anxiety symptoms, quality of life, general distress, and stress). Subgroup analyses were performed using Comprehensive Meta-Analysis (version 3; Bio-stat Inc) under a mixed effects model, which pools studies within a subgroup using a random effects model but tests for statistically significant differences between subgroups using fixed effects models [72]. As avatar does not fit the definition of the self-guided intervention, we pooled the effect sizes of the chatbot, ECA, and CA in VR to show the effect of self-guided CAIs in subgroup analysis.

Univariate random effects meta-regression used residual restricted maximum likelihood to measure between-study variance (τ^2^) with a Knapp-Hartung modification as recommended models [73].

We applied different methods to examine publication bias (funnel plot, Begg and Mazumdar rank correlation test [74], Egger regression test [75], and Duval and Tweedie trim-and-fill procedure [76]).

## Results

**Figure 1:**
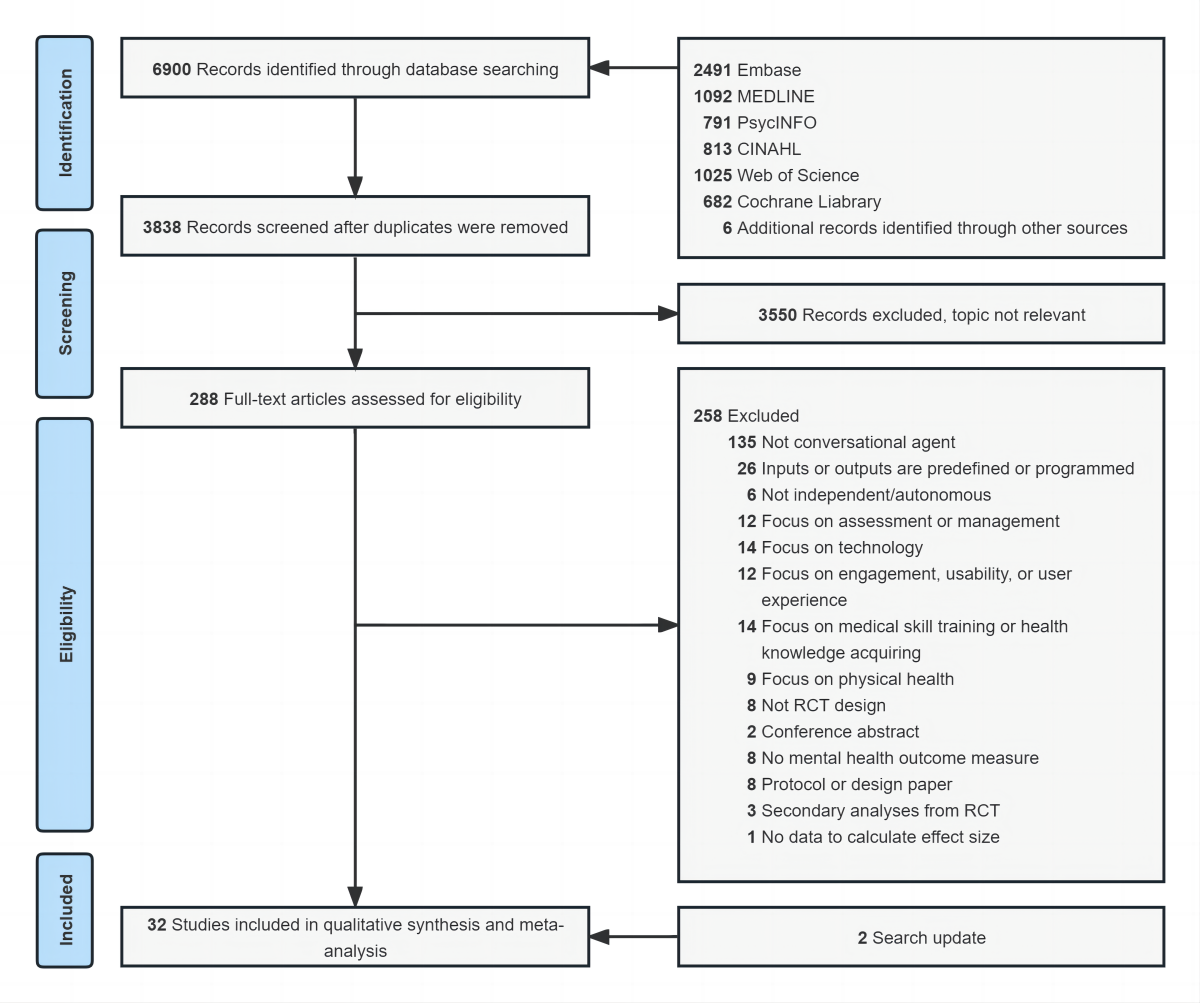
PRISMA (Preferred Reporting Items for Systematic Reviews and Meta-Analyses) study flow diagram. RCT: randomized controlled trial.

The details of the selection process are presented in a PRISMA flow diagram (Figure 1).

### Study Characteristics

The 32 RCTs with 26 CAs were included, involving 6089 participants, with sample sizes ranging from 19 to 2668 (Table S2 in Multimedia Appendix 1). The average age was 36.32 (SD 13.44; range 21.40-71.47) years, and 75.41% (2922/3875) of the participants were women from the reported data. A total of 16 studies with CAs were classified as chatbot [28-43], 6 studies with CAs classified as ECA [44-49], 5 studies with CAs classified as CA in VR [50-54], and 5 studies with CAs classified as avatar [27,55-58].

Among the included studies, 9 were conducted in the United States; 6 were conducted in the United Kingdom; 3 were conducted in Sweden; 2 each conducted in Japan, Korea, Switzerland, and Canada; and 1 each conducted in Argentina, China, Ireland, the Netherlands, Germany, and New Zealand. A total of 8 studies recruited clinical samples, 14 recruited symptomatic samples, and 10 recruited general samples. In addition, 17 studies were based on cognitive behavioral therapy (CBT), and 15 studies were based on other theories such as patient-centered therapy, acceptance and commitment therapy, method of levels therapy, and problem-solving treatment. A total of 18 studies supported personalization and tailoring, and 14 studies did not support personalization and tailoring. Moreover, 18 studies supported emotional and empathic responses, and 14 studies did not support emotional and empathic responses. In total, 15 studies provided automatic reminders to engage, and 17 studies did not provide automatic reminders to engage. Nine studies directly aimed at depressive symptoms, 11 studies directly aimed at generalized anxiety symptoms, 6 studies directly aimed at specific anxiety symptoms, 2 studies directly aimed at quality of life, 5 studies directly aimed at general distress, 2 studies directly aimed at stress, and 4 studies directly aimed at other outcomes. Eleven studies had an intervention length of 0 to 4 weeks, 10 studies had an intervention length of 5 to 8 weeks, and 11 studies had an intervention length of >9 weeks. Seventeen studies had no follow-up, 7 studies had a follow-up length of 0 to 8 weeks, and 8 studies had a follow-up length of ≥9 weeks. Fourteen studies used passive controls, 8 studies used information or attentional controls, and 10 studies used active controls.

Attrition rates between baseline and postintervention measures were reported in 31 studies and varied widely from no attrition to 82.98% (2214/2668) of participants. More dropouts were from the control condition (2081/3583, 58.08%) than from the intervention condition (740/1888, 39.19%). The ration of the interaction reported in 16 studies ranged from 0.57 to 6.43 (mean 4.48, SD 2.11) minutes per day. Most studies (14/17, 82%) have reported good acceptability and usability of CAIs. Fifteen studies reported user experience, of which 8 performed thematic analysis on the feedback of participants.

### Risk of Bias

Interrater reliability suggested substantial agreement between the raters for 7 domains of the Cochrane Collaboration tool (Cohen κ=0.86, 0.79, 0.67, 0.89, 0.93, 0.75, and 0.83, respectively). Eight studies were assessed as having a low risk of bias, 11 studies had some risk of bias, and 13 studies had a high risk of bias (Figures S1 and S2 in Multimedia Appendix 1).

The funnel plot of short-term effects (Figure S3 in Multimedia Appendix 1) and long-term effects (Figure S4 in Multimedia Appendix 1) and the results of Begg and Egger tests performed well on most mental health outcomes (Table S3 in Multimedia Appendix 1).

### Efficacy

#### Depressive Symptoms

**Figure 2:**
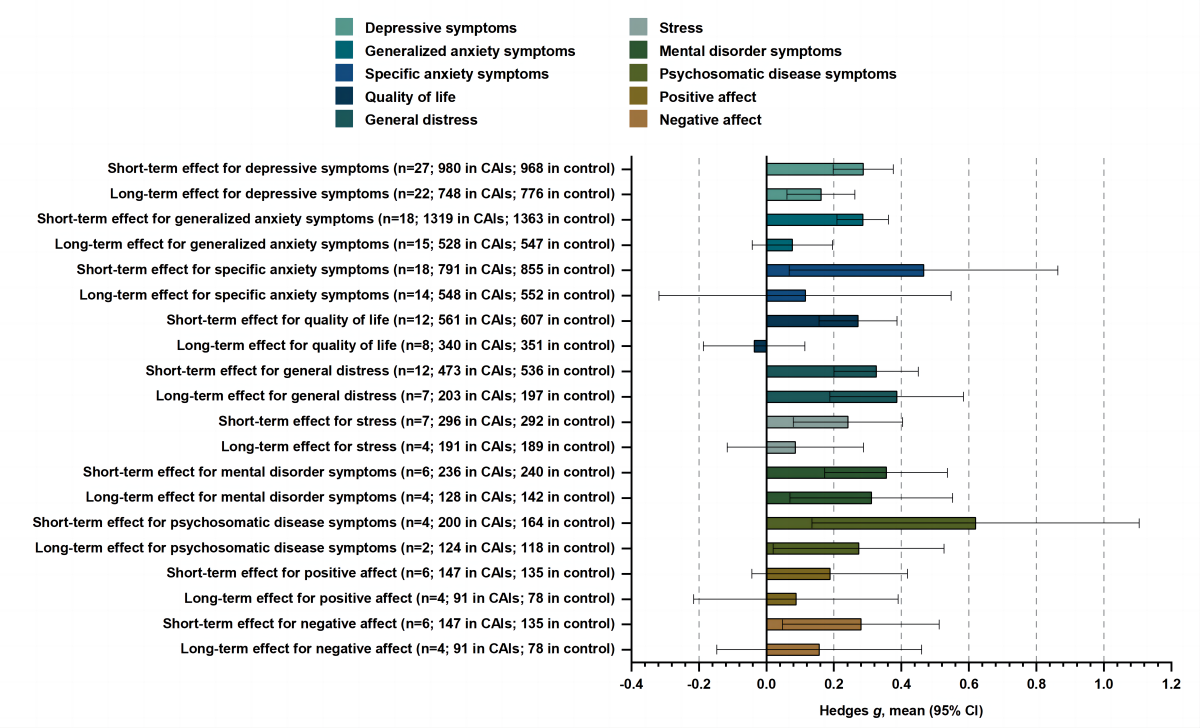
Overall effect analyses with short- and long-term efficacy of CAIs for mental health problems. Hedges g scores (mean and 95% CI) are given (positive values indicate better performance among individuals at CAIs vs control individuals), along with the number of comparisons (n) included and sample size. CAI: conversational agent intervention.

**Figure 3:**
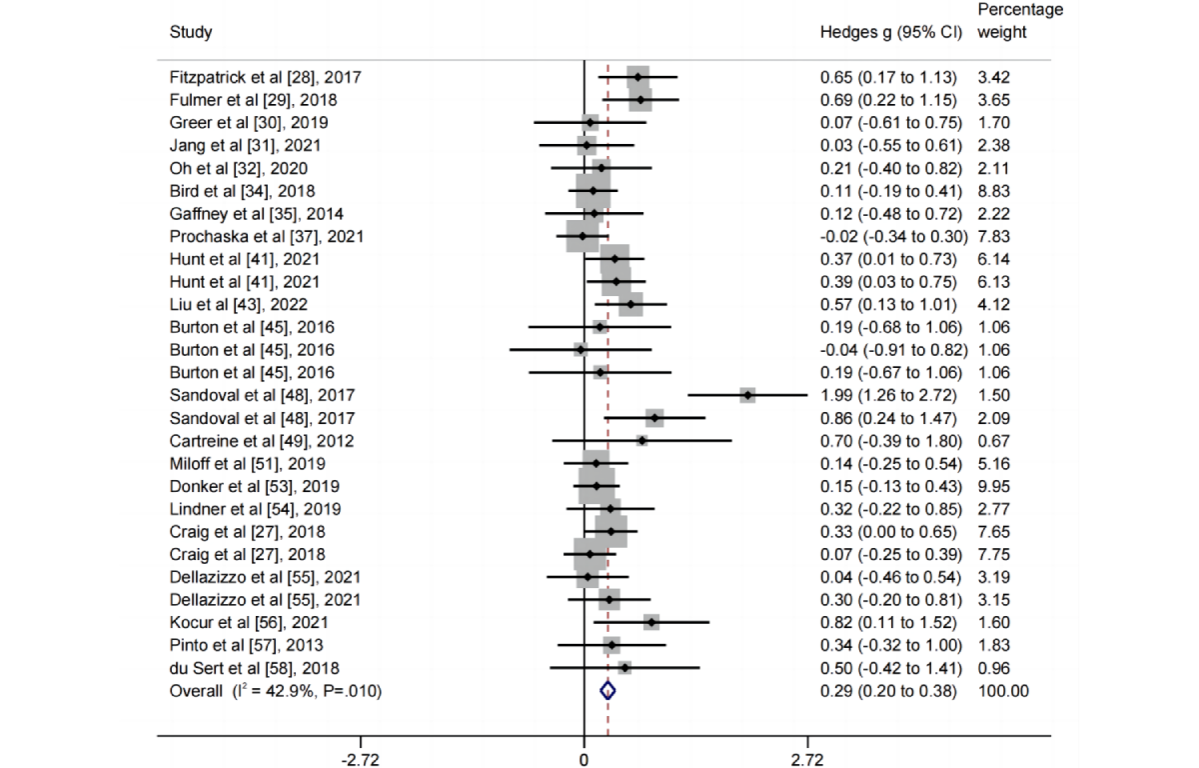
Forest plot for the short-term effects of conversational agent interventions on depressive symptoms.

The pooled effect size for the 27 postintervention comparisons between CAIs and control conditions on depressive symptoms was g=0.29 (95% CI 0.20-0.38), with moderate heterogeneity (*I*^2^=42.90%, 95% CI 9.79%-63.86%; Figures 2 and 3; Table 1). This effect size was slightly smaller after adjusting for any potential publication bias (*g*=0.26) and slightly larger when restricting the analyses to trials with a low risk of bias (*g*=0.32; Table 1). The pooled effect size of long-term efficacy for the 22 follow-up comparisons between CAIs and control conditions on depressive symptoms was *g*=0.16 (95% CI 0.06-0.26), with low heterogeneity (*I*^2^=8.05%, 95% CI 0.00%-42.12%; Figure 2; Table 1; Figure S5 in Multimedia Appendix 1).

**Figure 4:**
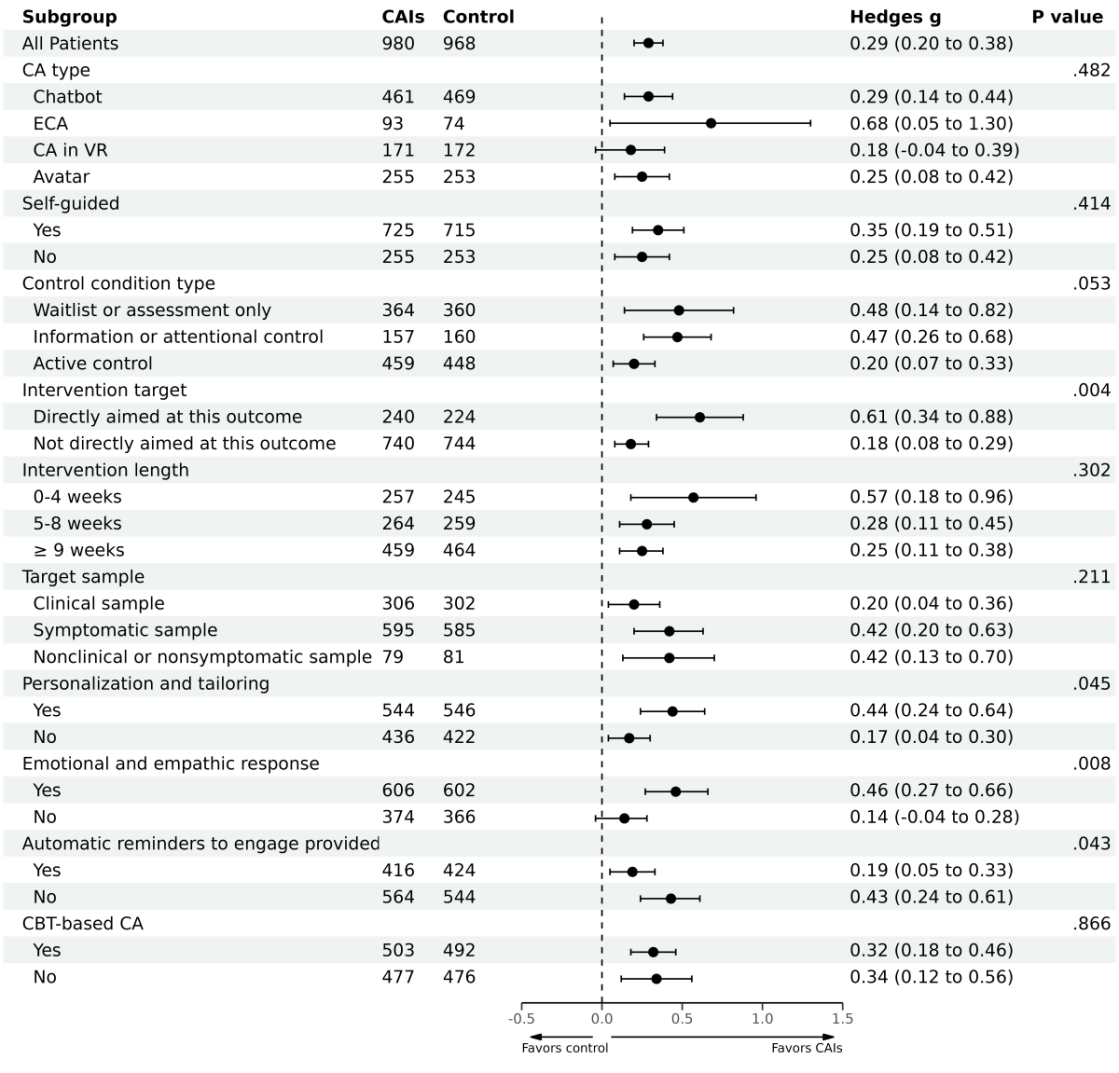
Subgroup analyses of efficacy of CAIs for depressive symptoms. *P* value represents the significance of the Q test. CA: conversational agent; CAI: conversational agent intervention; CBT: cognitive behavioral therapy; ECA: embodied conversational agent; VR: virtual reality; WL: waitlist.

The subgroup analyses revealed 5 statistically significant moderators (Figure 4; Table 1). Studies that directly aimed at depressive symptoms produced larger effect sizes than those that did not (*P*=.004). Studies with a follow-up length between 0 and 8 weeks or between 9 and 16 weeks produced larger effect sizes than those with a follow-up length of ≥17 weeks (*P*=.049). Studies that supported personalization and tailoring produced larger effect sizes than those that did not (*P*=.045). Studies that supported emotional and empathic responses produced larger effect sizes than those that did not (*P*=.008). Studies that provided automatic reminders to engage produced smaller effect sizes than those that did not (*P*=.04).

Meta-regression analyses revealed a statistically significant effect of dosage (*b*=0.160, 95% CI 0.014-0.306; *P*=.04) on the pooled effect size (Table 2; Figure S6 in Multimedia Appendix 1).

**Table 1:** Meta-analysis of efficacy of conversational agent interventions for depressive and generalized anxiety symptoms.

				Depressive symptoms										Generalized anxiety symptoms							
				Value, n^a^		*g* (95% CI)		*I*^2^ (%; 95% CI)		*P* value^b^			Value, n^a^			*g* (95% CI)		*I*^2^ (%; 95% CI)		*P* value^b^	
**Overall effect analysis**											N/A^c^										N/A
	Short-term effect			27		0.29 (0.20 to 0.38)^d^		42.90 (9.79 to 63.86)					18			0.29 (0.21 to 0.36)^d^		27.32 (0.00 to 58.94)			
	Adjusted for publication bias			30		0.26 (0.11 to 0.41)^e^		N/A					18			0.29 (0.21 to 0.36)^d^		N/A			
	Long-term effect			22		0.16 (0.06 to 0.26)^e^		8.05 (0.00 to 42.12)					15			0.08 (−0.04 to 0.20)		0.00 (0.00 to 53.61)			
	Adjusted for publication bias			23		0.15 (0.05 to 0.25)^e^		N/A					19			0.00 (−0.11 to 0.11)		N/A			
**Sensitivity analysis**											N/A										N/A
	One effect size per study (largest)			21		0.36 (0.21 to 0.50)^d^		50.44 (18.09 to 70.01)					17			0.28 (0.19 to 0.36)^d^		30.76 (0.00 to 61.45)			
	One effect size per study (smallest)			21		0.26 (0.17 to 0.36)^d^		22.48 (0.00 to 54.46)					17			0.24 (0.15 to 0.32)^d^		10.92 (0.00 to 47.69)			
	Low risk of bias only (all criteria met)			7		0.32 (0.18 to 0.46)^d^		6.07 (0.00 to 41.68)					5			0.18 (0.01 to 0.35)^f^		0.00 (0.00 to 79.20)			
**Subgroup analyses**																					
	**Conversational agent type**										.48										.46
		Chatbot	11		0.29 (0.14 to 0.44)^d^		25.76 (0.00 to 63.21)					13			0.29 (0.17 to 0.41)^d^		31.89 (0.00 to 64.80)				
		Embodied conversational agent	6		0.68 (0.05 to 1.30)^f^		71.65 (34.28 to 87.77)					0			—^g^		—				
		Conversational agent in virtual reality	3		0.18 (−0.04 to 0.39)		0.00 (0.00 to 89.60)					3			0.23 (0.02 to 0.46)^f^		11.24 (0.00 to 51.16)				
		Avatar	7		0.25 (0.08 to 0.42)^e^		0.00 (0.00 to 70.81)					2			0.10 (−0.17 to 0.37)		0.00				
	**Self-guided**										.41										.22
		Yes	20		0.35 (0.19 to 0.51)^d^		52.85 (0.22 to 0.72)					16			0.28 (0.18 to 0.38)^d^		26.74 (0.00 to 59.85)				
		No	7		0.25 (0.08 to 0.42)^e^		0.00 (0.00 to 70.81)					2			0.10 (−0.17 to 0.37)		0.00				
	**Control condition type**										.05										.06
		Waitlist or assessment only	8		0.48 (0.14 to 0.82)^e^		76.67 (53.55 to 88.29)					6			0.35 (0.25 to 0.44)^d^		0.00 (0.00 to 74.63)				
		Information or attentional control	6		0.47 (0.26 to 0.68)^d^		0.00 (0.00 to 74.63)					6			0.27 (0.01 to 0.54)^f^		38.56 (0.00 to 75.59)				
		Active control	13		0.20 (0.07 to 0.33)^e^		0.00 (0.00 to 56.60)					6			0.12 (−0.05 to 0.28)		0.00 (0.00 to 74.63)				
	**Intervention target**										*.004* ^h^										*.03*
		Directly aimed at this outcome	12		0.61 (0.34 to 0.88)^d^		49.82 (2.62 to 74.14)					12			0.33 (0.22 to 0.43)^d^		18.95 (0.00 to 57.96)				
		Not directly aimed at this outcome	15		0.18 (0.08 to 0.29)^d^		0.00 (0.00 to 53.61)					6			0.11 (−0.05 to 0.28)		0.00 (0.00 to 74.63)				
	**Intervention length (weeks)**										.30										.44
		0-4	8		0.57 (0.18 to 0.96)^e^		75.12 (49.90 to 87.64)					4			0.15 (−0.10 to 0.39)		25.32 (0.00 to 71.30)				
		5-8	10		0.28 (0.11 to 0.45)^e^		0.00 (0.00 to 62.37)					6			0.25 (0.07 to 0.49)^f^		33.15 (0.00 to 73.08)				
		≥9	9		0.25 (0.11 to 0.38)^d^		2.12 (0.00 to 23.51)					8			0.32 (0.21 to 0.43)^d^		15.57 (0.00 to 58.54)				
	**Follow-up length^i^ (weeks)**										*.049*										.74
		0-8	5		0.30 (−0.01 to 0.61)		29.97 (0.00 to 72.95)					4			0.05 (−0.21 to 0.32)		0.00 (0.00 to 84.69)				
		9-16	8		0.26 (0.10 to 0.41)^e^		0.00 (0.00 to 67.58)					5			0.15 (−0.03 to 0.34)		0.00 (0.00 to 79.20)				
		≥17	9		0.01 (−0.15 to 0.16)		0.00 (0.00 to 64.80)					6			0.05 (−0.18 to 0.27)		23.24 (0.00 to 67.22)				
	**Target sample**										.21										*.005*
		Clinical sample	10		0.20 (0.04 to 0.36)^f^		0.00 (0.00 to 62.37)					4			0.04 (−0.18 to 0.27)		0.00 (0.00 to 84.69)				
		Symptomatic sample	13		0.42 (0.20 to 0.63)^d^		66.43 (39.76 to 81.29)					10			0.21 (0.09 to 0.34)^e^		2.65 (0.00 to 27.71)				
		Nonclinical or nonsymptomatic sample	4		0.42 (0.13 to 0.70)^e^		0.00 (0.00 to 84.69)					4			0.40 (0.29 to 0.51)^d^		0.00 (0.00 to 84.69)				
	**Personalization and tailoring**										*.05*										*.04*
		Yes	17		0.44 (0.24 to 0.64)^d^		59.96 (30.18 to 76.04)					12			0.33 (0.21 to 0.44)^d^		21.36 (0.00 to 59.62)				
		No	10		0.17 (0.04 to 0.30)^e^		0.00 (0.00 to 62.37)					6			0.13 (−0.02 to 0.28)		0..00 (0.00 to 74.63)				
	**Emotional and empathic responses**										*.008*										*.02*
		Yes	16		0.46 (0.27 to 0.66)^d^		60.63 (32.01 to 77.20)					10			0.34 (0.23 to 0.44)^d^		18.19 (0.00 to 59.10)				
		No	11		0.14 (−0.04 to 0.28)		0.00 (0.00 to 60.23)					8			0.12 (−0.03 to 0.27)		0.00 (0.00 to 67.58)				
	**Automatic reminders to engage provided**										*.04*										.22
		Yes	11		0.19 (0.05 to 0.33)^e^		5.89 (0.00 to 41.41)					10			0.20 (0.04 to 0.35)^f^		21.20 (0.00 to 61.32)				
		No	16		0.43 (0.24 to 0.61)^d^		51.26 (13.59 to 72.51)					8			0.32 (0.20 to 0.43)^d^		20.00 (0.00 to 62.47)				
	**Cognitive behavioral therapy–based conversational agent**										.87										.93
		Yes	14		0.32 (0.18 to 0.46)^d^		15.14 (0.00 to 53.48)					9			0.25 (0.10 to 0.40)^e^		13.82 (0.00 to 55.85)				
		No	13		0.34 (0.12 to 0.56)^e^		60.05 (26.57 to 78.26)					9			0.26 (0.12 to 0.40)^d^		41.55 (0.00 to 73.06)				

^a^Number of comparisons.

^b^*P* value represents the significance of the *Q* test.

^c^N/A: not applicable.

^d^*P*<.001.

^e^*P*<.01.

^f^*P*<.05.

^g^Missing data.

^h^Italicized values indicate statistically significant differences.

^i^Follow-up length is a moderator of long-term effects, whereas the other variables are moderators of short-term effects.

**Table 2:** Meta-regression of efficacy of conversational agent interventions for primary mental health outcomes.

Dependent and covariate		Coefficient (95% CI)	SE	*P* value
**Depressive symptoms**				
	Age	−0.011 (−0.027 to 0.005)	0.008	.18
	Gender	0.080 (−0.712 to 0.872)	0.385	.84
	Dose^a^	0.160 (0.014 to 0.306)	0.067	*.04* ^b^
**Generalized anxiety symptoms**				
	Age	−0.007 (−0.022 to 0.008)	0.007	.33
	Gender	0.438 (0.029 to 0.847)	0.193	*.04*
	Dose^a^	0.067 (0.006 to 0.127)	0.025	*.04*
**Specific anxiety symptoms**				
	Age	0.084 (0.014 to 0.154)	0.033	*.01*
	Gender	−3.414 (−6.118 to −0.710)	1.276	*.02*
	Dose^a^	0.186 (−0.106 to 0.474)	0.122	.10
**General distress**				
	Age	−0.003 (−0.014 to 0.008)	0.005	.50
	Gender	−1.078 (−4.097 to 1.940)	1.355	.45
	Dose^a^	0.021 (−0.744 to 0.786)	0.178	.92
**Quality of life**				
	Age	0.008 (−0.017 to 0.032)	0.011	.52
	Gender	0.151 (−0.975 to 1.277)	0.505	.71
	Dose^a^	0.102 (−0.429 to 0.633)	0.167	.54
**Stress**				
	Age	0.014 (−0.007 to 0.035)	0.008	.16
	Gender	0.251 (−1.498 to 1.999)	0.680	.73
	Dose^a^	−0.207 (−16.303 to 15.890)	1.267	.90

^a^Average duration of interaction with conversational agent per day.

^b^Italicized values indicate statistically significant differences.

#### Generalized Anxiety Symptoms

**Figure 5:**
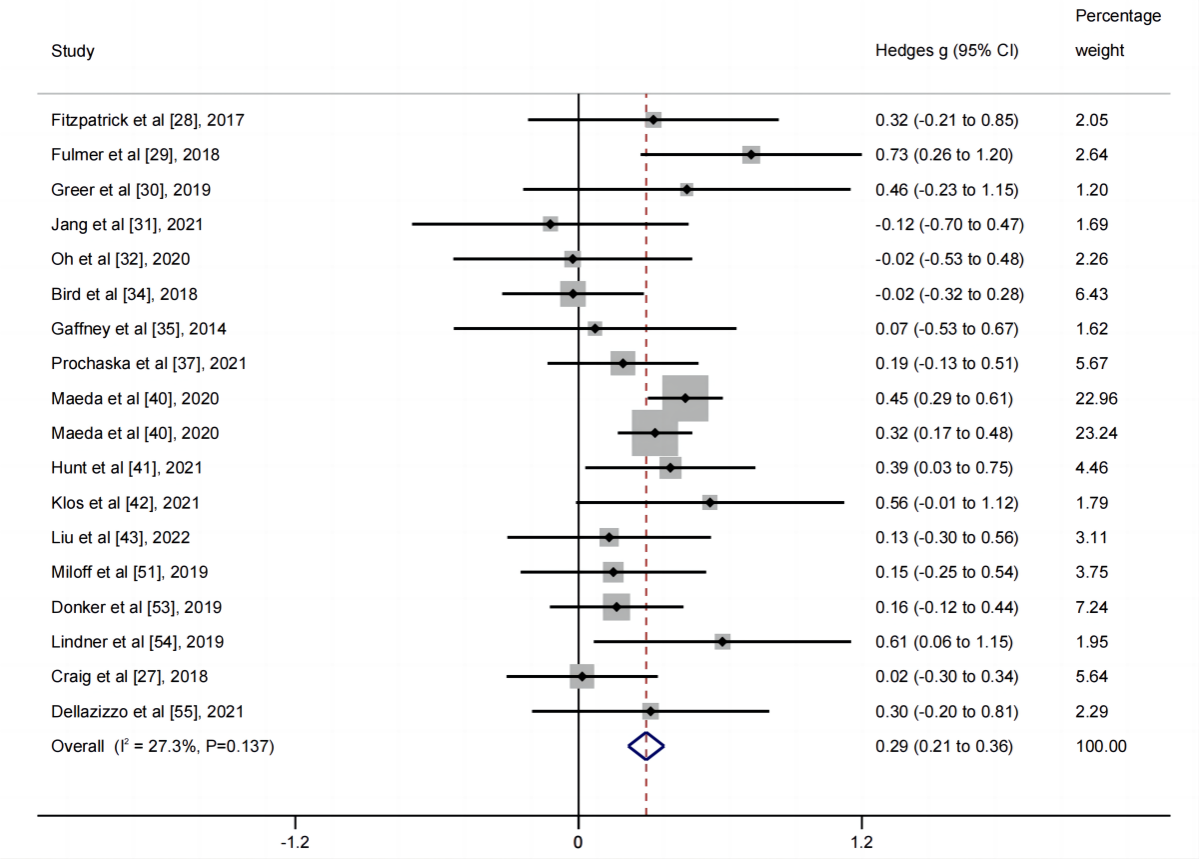
Forest plot for the short-term effects of conversational agent interventions on generalized anxiety symptoms.

The pooled effect size for the 18 postintervention comparisons was *g*=0.29 (95% CI 0.21-0.36), with moderate heterogeneity (*I*^2^=27.32%, 95% CI 0.00%-58.94%; Figures 2 and 5; Table 1). It remained significant across all sensitivity analyses (Table 1). The pooled effect size of long-term efficacy was *g*=0.08 (95% CI −0.04 to 0.20), with low heterogeneity (*I*^2^=0.00%, 95% CI 0.00%-53.61%; Figure 2; Table 1; Figure S7 in Multimedia Appendix 1).

**Figure 6:**
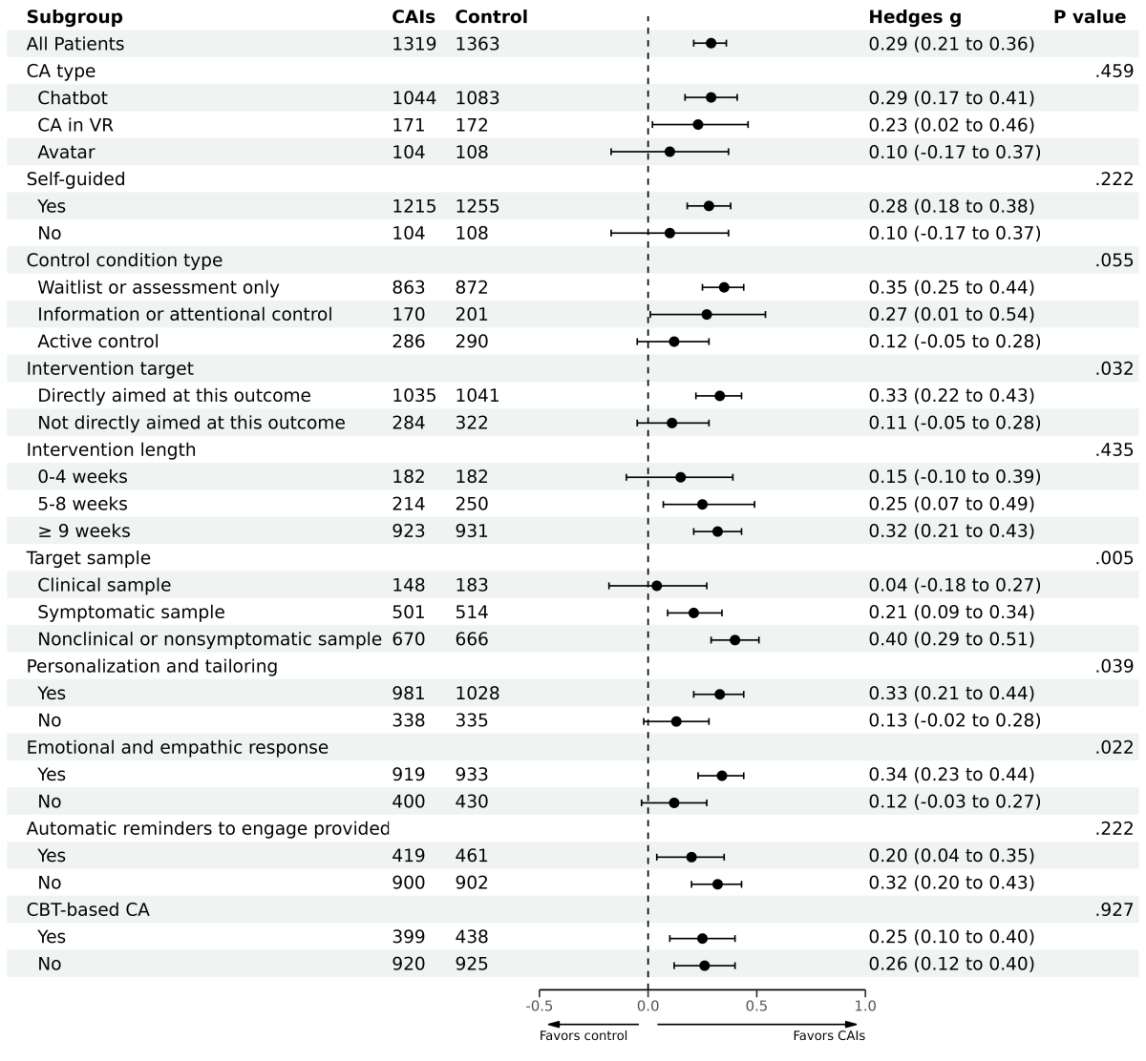
Subgroup analyses of efficacy of CAIs for generalized anxiety symptoms. *P* value represents the significance of the Q test. CA: conversational agent; CAI: conversational agent intervention; CBT: cognitive behavioral therapy; ECA: embodied conversational agent; VR: virtual reality; WL: waitlist.

The subgroup analyses revealed 4 statistically significant moderators (Figure 6; Table 1). Larger effect sizes were found in studies that directly aimed at generalized anxiety symptoms (*P*=.03), that with symptomatic sample or general sample (*P*=.005), that supported personalization and tailoring (*P*=.04), and that supported emotional and empathic responses (*P*=.02).

Meta-regression analyses revealed statistically significant effects of gender (*b*=0.448, 95% CI 0.024-0.873; *P*=.04) and dosage (*b*=0.067, 95% CI 0.006-0.127; *P*=.04) on the pooled effect size (Table 2; Figure S6 in Multimedia Appendix 1).

#### Specific Anxiety Symptoms

The pooled effect size for the 18 postintervention comparisons was *g*=0.47 (95% CI 0.07-0.86), with high heterogeneity (*I*^2^=93.17%, 95% CI 90.62%-95.03%; Figure 2; Table 3; Figure S8 in Multimedia Appendix 1). It did not remain significant when compared with the smallest effect size and when restricting the analyses to trials with a low risk of bias (Table 3). The pooled effect size of long-term efficacy was *g*=0.11 (95% CI −0.32 to 0.55), with high heterogeneity (*I*^2^=91.82%, 95% CI 88.01%-94.42%; Figure 2; Table 3; Figure S9 in Multimedia Appendix 1).

Subgroup analyses revealed 5 statistically significant moderators (Table 3; Figure S10 in Multimedia Appendix 1). Larger effect sizes were found in studies with WL or assessment controls (*P*<.001), longer intervention lengths (5-8 weeks; *P*<.001), shorter follow-up length (0-8 weeks; *P*<.001), CBT-based CAs (*P*=.008), and studies that supported emotional and empathic responses (*P*<.001).

Meta-regression analyses revealed statistically significant effects of age (*b*=0.084, 95% CI 0.014-0.154; *P*=.02) and gender (*b*=−3.414, 95% CI −6.118 to −0.710; *P*=.02) on the pooled effect size (Table 2; Figure S6 in Multimedia Appendix 1).

**Table 3:** Meta-analysis of efficacy of conversational agent interventions for specific anxiety symptoms and quality of life.

				Specific anxiety symptoms											Quality of life											
				Value, n^a^		g (95% CI)		I^2^ (%; 95% CI)		*P* value^b^				Value, n^a^				g (95% CI)			I^2^ (%, 95% CI)			*P* value^b^		
**Overall effect analysis**												N/A^c^													N/A	
	Short-term effect			18		0.47 (0.07 to 0.86)^d^		93.17 (90.62 to 95.03)						12				0.27 (0.16 to 0.39)^e^			44.16 (0.00 to 71.54)					
	Adjusted for publication bias			18		0.47 (0.07 to 0.86)^d^		N/A						15				0.18 (0.07 to 0.29)^e^			N/A					
	Long-term effect			14		0.11 (−0.32 to 0.55)		91.82 (88.01 to 94.42)						8				−0.04 (−0.19 to 0.11)			24.17 (0.00 to 64.28)					
	Adjusted for publication bias			14		0.11 (−0.23 to 0.67)		N/A						8				−0.04 (−0.19 to 0.11)			N/A					
**Sensitivity analysis**												N/A														N/A
	One effect size per study (largest)			6		0.88 (0.35 to 1.41)^e^		88.44 (77.37 to 94.09)						11				0.27 (0.16 to 0.39)^e^			49.21 (0.00 to 74.60)					
	One effect size per study (smallest)			6		0.22 (−0.77 to 1.20)		96.64 (94.64 to 97.89)						11				0.27 (0.15 to 0.39)^e^			49.21 (0.00 to 74.60)					
	Low risk of bias only (all criteria met)			5		0.54 (−0.67 to 1.75)		97.43 (95.84 to 98.40)						3				0.38 (−0.14 to 0.89)			83.54 (50.25 to 94.55)					
**Subgroup analyses**																										
	**CA^f^ type**										.09														*.04* ^g^	
		Chatbot	2		0.03 (−0.33 to 0.39)		0.00						5				0.54 (0.26 to 0.83)^e^			29.70 (0.00 to 72.79)						
		Embodied CA	0		—^h^		—						2				0.21 (0.03 to 0.40)^d^			0.00						
		CA in virtual reality	16		0.52 (0.08 to 0.96)^d^		93.78 (91.36 to 95.52)						2				−0.08 (−0.40 to 0.24)			0.00						
		Avatar	0		—		—						3				0.24 (−0.02 to 0.50)			0.00 (0.00 to 89.60)						
	**Self-guided**										—														.69	
		Yes	18		0.47 (0.07 to 0.86)^d^		93.17 (90.62 to 95.03)						9				0.31 (0.08 to 0.54)^d^			55.10 (5.08 to 78.76)						
		No	0		—		—						3				0.24 (−0.02 to 0.50)			0.00 (0.00 to 89.60)						
	**Control condition type**										*<.001*														.07	
		Waitlist or assessment only	10		0.99 (0.63 to 1.36)^e^		86.59 (77.26 to 92.09)						6				0.44 (0.16 to 0.71)^i^			56.67 (0.00 to 82.54)						
		Information or attentional control	2		0.03 (−0.33 to 0.39)		0.00						0				—			—						
		Active control	6		−0.30 (−0.73 to 0.14)		80.45 (57.78 to 90.96)						6				0.13 (−0.07 to 0.32)			0.00 (0.00 to 74.63)						
	**Intervention target**										—														.49	
		Directly aimed at this outcome	18		0.47 (0.07 to 0.86)^d^		93.17 (90.62 to 95.03)						3				0.21 (0.04 to 0.39)^d^			0.00 (0.00 to 89.60)						
		Not directly aimed at this outcome	0		—		—						9				0.32 (0.07 to 0.58)^d^			57.51 (10.85 to 79.75)						
	**Intervention length (weeks)**										*<.001*														.36	
		0-4	9		−0.09 (−0.46 to 0.28)		82.68 (68.48 to 90.48)						5				0.10 (−0.16 to 0.39)			11.11 (0.00 to 53.67)						
		5-8	9		1.01 (0.61 to 1.42)^e^		87.81 (79.00 to 92.93)						1				0.43 (−0.44 to 1.31)			N/A						
		≥9	0		—		—						6				0.37 (0.13 to 0.62)^i^			61.24 (5.30 to 84.14)						
	**Follow-up length^j^ (weeks)**										*<.001*														.54	
		0-8	2		1.96 (1.62 to 2.30)^e^		0.00						0				—			—						
		9-16	3		−0.22 (−0.86 to 0.42)		87.02 (62.98 to 95.45)						3				0.06 (−0.33 to 0.44)			59.50 (0.00 to 88.46)						
		≥17	9		−0.19 (−0.36 to −0.01)^d^		12.25 (0.00 to 53.96)						6				−0.08 (−0.28 to 0.12)			4.08 (0.00 to 32.89)						
	**Target sample**										.52														.34	
		Clinical sample	8		0.45 (0.18 to 0.71)^e^		60.50 (14.22 to 81.81)						3				0.21 (−0.05 to 0.47)			0.00 (0.00 to 89.60)						
		Symptomatic sample	7		0.59 (−0.31 to 1.49)		97.34 (96.05 to 98.21)						5				0.49 (0.10 to 0.88)^d^			67.48 (15.79 to 87.44)						
		Nonclinical or nonsymptomatic sample	3		0.23 (−0.09 to 0.55)		0.00 (0.00 to 89.60)						4				0.17 (0.01 to 0.34)^d^			0.00 (0.00 to 84.69)						
	**Personalization and tailor**										.12														.25	
		Yes	5		0.14 (−0.10 to 0.38)		0.00 (0.00 to 79.20)						6				0.18 (−0.04 to 0.39)			0.00 (0.00 to 74.63)						
		No	13		0.59 (0.07 to 1.10)^d^		94.93 (92.84 to 96.41)						6				0.39 (0.10 to 0.67)^i^			65.88 (18.31 to 85.75)						
	**Emotional and empathic responses**										*<.001*														*.047*	
		Yes	2		1.96 (1.63 to 2.30)^e^		0.00						8				0.43 (0.20 to 0.66)^e^			36.53 (0.00 to 71.95)						
		No	16		0.28 (−0.09 to 0.65)		91.27 (87.44 to 93.93)						4				0.14 (−0.03 to 0.30)			1.61 (0.00 to 14.13)						
	**Automatic reminders to engage provided**										.53														.41	
		Yes	4		0.67 (0.03 to 1.31)^d^		90.84 (79.63 to 95.88)						3				0.42 (0.10 to 0.74)^d^			0.00 (0.00 to 89.60)						
		No	14		0.41 (−0.07 to 0.89)		93.17 (90.18 to 95.25)						9				0.26 (0.04 to 0.48)^d^			55.83 (6.81 to 79.06)						
	**Cognitive behavioral therapy** **–** **based CA**										*.008*														.08	
		Yes	6		1.09 (0.50 to 1.68)^e^		91.78 (84.88 to 95.54)						7				0.43 (0.18 to 0.69)^e^			48.28 (0.00 to 78.14)						
		No	12		0.15 (−0.23 to 0.53)		87.54 (80.09 to 92.20)						5				0.12 (−0.10 to 0.35)			18.03 (0.00 to 63.71)						

^a^Number of comparisons.

^b^*P* value represents the significance of the *Q* test.

^c^N/A: not applicable.

^d^*P*<.05.

^e^*P*<.001.

^f^CA: conversational agent.

^g^Italicized values indicate statistically significant differences.

^h^Missing data.

^i^*P*<.01.

^j^Follow-up length is a moderator of long-term effects, whereas the other variables are moderators of short-term effects.

#### Quality of Life or Well-being

The pooled effect size for the 12 postintervention comparisons was *g*=0.27 (95% CI 0.16 to 0.39), with moderate heterogeneity (*I*^2^=44.16%, 95% CI 0.00%-71.54%; Figure 2; Table 3; Figure S11 in Multimedia Appendix 1). It did not remain significant when restricting the analyses to trials with a low risk of bias (Table 3). The pooled effect size of long-term efficacy was *g*=−0.04 (95% CI −0.19 to 0.11), with low heterogeneity (*I*^2^=24.17%, 95% CI 0.00%-64.28%; Figure 2; Table 3; Figure S12 in Multimedia Appendix 1).

The subgroup analyses revealed 2 statistically significant moderators (Table 3; Figure S13 Multimedia Appendix 1). Larger effect sizes were found in studies with chatbot (*P*=.04) and those that supported emotional and empathic responses (*P*=.047).

Meta-regression analyses revealed no statistically significant results (Table 2).

#### General Distress

The pooled effect size for the 12 postintervention comparisons was *g*=0.33 (95% CI 0.20-0.45), with low heterogeneity (*I*^2^=6.93%, 95% CI 0.00%-43.77%; Figure 2; Table 4; Figure S14 in Multimedia Appendix 1). It remained significant across all sensitivity analyses (Table 4). The pooled effect size of long-term efficacy was *g*=0.39, with low heterogeneity (*I*^2^=0.00%, 95% CI 0.00%-70.81%; Figure 2; Table 4; Figure S15 in Multimedia Appendix 1).

The subgroup analyses revealed 2 statistically significant moderators (Table 4; Figure S16 in Multimedia Appendix 1). Larger effect sizes were found in studies that supported personalization and tailoring (*P*=.002) and that supported emotional and empathic responses (*P*=.03).

Meta-regression analyses revealed no statistically significant results (Table 2).

**Table 4:** Meta-analysis of efficacy of conversational agent interventions for general distress and stress.

					General distress															Stress														
					Value, n^a^			*g* (95% CI)			*I*^2^ (%; 95% CI)			*P* value^b^					Value, n^a^					*g* (95% CI)				*I*^2^ (%; 95% CI)			*P* value^b^			
**Overall effect analysis**																N/A^c^																		N/A
	Short-term effect			12			0.33 (0.20 to 0.45)^d^			6.93 (0.00 to 43.77)								7					0.24 (0.08 to 0.41)^e^				39.10 (0.00 to 74.39)							
	Adjusted for publication bias			15			0.27 (0.15 to 0.39)^d^			N/A								8					0.27 (0.05 to 0.50)^f^				N/A							
	Long-term effect			7			0.39 (0.19 to 0.59)^d^			0.00 (0.00 to 70.81)								4					0.09 (−0.12 to 0.29)				0.00 (0.00 to 84.69)							
	Adjusted for publication bias			7			0.39 (0.19 to 0.59)^d^			N/A								4					0.09 (−0.12 to 0.24)				N/A							
**Sensitivity analysis**																N/A																		N/A
	One effect size per study (largest)			8			0.31 (0.18 to 0.45)^d^			14.92 (0.00 to 57.85)								7					0.24 (0.08 to 0.41)^e^				39.10 (0.00 to 74.39)							
	One effect size per study (smallest)			8			0.28 (0.15 to 0.41)^d^			0.00 (0.00 to 67.58)								7					0.24 (0.08 to 0.41)^e^				39.10 (0.00 to 74.39)							
	Low risk of bias only (all criteria met)			1			0.44 (0.04 to 0.84)^f^			—^g^								2					0.33 (0.09 to 0.57)^e^				62.55 (0.00 to 91.38)							
**Subgroup analyses**																																		
	**CA^h^ type**													.06																		.15		
		Chatbot	5			0.45 (0.19 to 0.71)^e^			31.46 (0.00 to 79.20)								5					0.24 (−0.02 to 0.49)				33.65 (0.00 to 74.92)								
		Embodied CA	5			0.22 (0.06 to 0.39)^e^			0.00 (0.00 to 79.20)								1					0.91 (0.21 to 1.61)^f^				—								
		CA in virtual reality	0			—			—								0					—				—								
		Avatar	2			0.80 (0.29 to 1.30)^e^			0.00								1					0.15 (−0.17 to 0.47)				—								
	**Self-guided**														.06																		.46	
		Yes	10			0.30 (0.17 to 0.42)^e^			0.00 (0.00 to 90.15)								6					0.32 (0.03 to 0.60)^f^				47.10 (0.00 to 79.04)								
		No	2			0.80 (0.29 to 1.30)^e^			0.00								1					0.15 (−0.17 to 0.47)				—								
	**Control condition type**														.77																		*.01* ^i^	
		Passive control	5			0.40 (0.11 to 0.69)^e^			39.45 (0.00 to 77.59)								3					0.62 (0.32 to 0.91)^d^				0.00 (0.00 to 89.60)								
		Information or attentional control	2			0.15 (−0.51 to 0.80)			0.00								1					0.09 (−0.50 to 0.68)				—								
		Active control	5			0.39 (0.17 to 0.61)^d^			8.82 (0.00 to 48.55)								3					0.08 (−0.12 to 0.29)				0.00 (0.00 to 89.60)								
	**Intervention target**														.11																		.05	
		Directly aimed at this outcome	10			0.43 (0.25 to 0.60)^d^			0.52 (0.00 to 8.00)								2					0.73 (0.22 to 1.25)^e^				0.00								
		Not directly aimed at this outcome	2			0.22 (0.04 to 0.40)^f^			0.00								5					0.19 (−0.02 to 0.40)				26.18 (0.00 to 70.60)								
	**Intervention length (weeks)**														.11																		.77	
		0-4	9			0.44 (0.25 to 0.64)^d^			13.77 (0.00 to 55.79)								4					0.30 (−0.10 to 0.70)				51.35 (0.00 to 83.92)								
		5-8	0			—			—								1					0.09 (−0.49 to 0.67)				—								
		≥9	3			0.23 (0.05 to 0.41)^f^			0.00 (0.00 to 89.60)								2					0.35 (−0.05 to 0.74)				62.55 (0.00 to 91.38)								
	**Follow-up length^j^ (weeks)**														—																		.44	
		0-8	7			0.39 (0.19 to 0.59)^d^			0.00 (0.00 to 70.81)								2					−0.11 (−0.48 to 0.27)				0.00								
		9-16	0			—			—								1					0.10 (−0.26 to 0.46)				—								
		≥17	0			—			—								1					0.22 (−0.11 to 0.54)				—								
	**Target sample**														.61																		.51	
		Clinical sample	4			0.56 (0.16 to 0.95)^e^			0.00 (0.00 to 84.69)								2					0.14 (−0.14 to 0.42)				0.00								
		Symptomatic sample	4			0.22 (−0.01 to 0.46)			0.00 (0.00 to 84.69)								4					0.34 (−0.04 to 0.73)				65.24 (0.00 to 88.19)								
		Nonclinical or nonsymptomatic sample	4			0.48 (0.17 to 0.78)^e^			52.73 (0.00 to 84.69)								1					0.53 (−0.23 to 1.29)				—								
	**Personalization and tailor**														*.002*																	.71		
		Yes	4			0.82 (0.48 to 1.16)^d^			0.00 (0.00 to 84.69)								2					0.21 (−0.09 to 0.51)				0.00								
		No	8			0.25 (0.12 to 0.38)^d^			0.00 (0.00 to 67.58)								5					0.29 (−0.02 to 0.61)				55.46 (0.00 to 83.54)								
	**Emotional and empathic responses**														*.03*																		*.04*	
		Yes	6			0.61 (0.33 to 0.89)^d^			3.68 (0.00 to 30.81)								4					0.46 (0.14 to 0.77)^e^				41.74 (0.00 to 80.38)								
		No	6			0.25 (0.11 to 0.39)^d^			0.00 (0.00 to 74.63)								3					0.04 (−0.20 to 0.29)				0.00 (0.00 to 89.60)								
	**Automatic reminders to engage provided**														.10																		.46	
		Yes	7			0.27 (0.07 to 0.46)^e^			0.00 (0.00 to 70.81)								4					0.20 (−0.15 to 0.54)				43.32 (0.00 to 81.01)								
		No	5			0.62 (0.25 to 0.98)^e^			56.69 (0.00 to 83.95)								3					0.37 (0.07 to 0.66)^f^				31.36 (0.00 to 76.73)								
	**Cognitive behavioral therapy** **–** **based CA**														.84																		.09	
		Yes	5			0.35 (0.10 to 0.60)^e^			15.29 (0.00 to 60.43)								3					0.44 (0.16 to 0.73)^e^				0.00 (0.00 to 89.60)								
		No	7			0.38 (0.18 to 0.58)^d^			11.07 (0.00 to 53.32)								5					0.13 (−0.10 to 0.36)				33.55 (0.00 to 74.87)								

^a^Number of comparisons.

^b^*P* value represents the significance of the *Q* test.

^c^N/A: not applicable.

^d^*P*<.001.

^e^*P*<.01.

^f^*P*<.05.

^g^Missing data.

^h^CA: conversational agent.

^i^Italicized values indicate statistically significant differences.

^j^Follow-up length is a moderator of long-term effects, whereas the other variables are moderators of short-term effects.

#### Stress

The pooled effect size for the 7 postintervention comparisons was *g*=0.24 (95% CI 0.08-0.41), with moderate heterogeneity (*I*^2^=39.10%, 95% CI 0.00%-74.39%; Figure 2; Table 4; Figure S17 in Multimedia Appendix 1). It remained significant across all sensitivity analyses (Table 4). The pooled effect size of long-term efficacy was *g*=0.09, with low heterogeneity (*I*^2^=0.00%, 95% CI 0.00%-84.69%; Figure 2; Table 4; Figure S18 in Multimedia Appendix 1).

Subgroup analyses revealed 2 statistically significant differences (Table 4; Figure S19 in Multimedia Appendix 1). Larger effect sizes were found in studies with WL or assessment controls (*P*=.01) and those that supported emotional and empathic responses (*P*=.04).

Meta-regression analyses revealed no statistically significant results (Table 2).

#### Other Outcomes

CAIs were significantly more effective than controls in improving mental disorder symptoms (*g*=0.36), psychosomatic disease symptoms (*g*=0.62), and negative affect (*g*=0.28), and the pooled effect sizes of long-term efficacy of mental disorder symptoms (*g*=0.31) and psychosomatic disease symptoms (*g*=0.27) were statistically significant (Figure 2; Table 5; Figures S20 and S21 in Multimedia Appendix 1).

**Table 5:** Meta-analysis of efficacy of conversational agent interventions for other outcomes.

Outcome measure		Value, n^a^	*g* (95% CI)	*I*^2^ (%; 95% CI)
**Mental disorder symptoms**				
	Short-term effect	6	0.36 (0.17 to 0.54)^b^	0.00 (00.00 to 74.63)
	Long-term effect	4	0.31 (0.07 to 0.55)^c^	0.00 (00.00 to 84.69)
**Psychosomatic disease symptoms**				
	Short-term effect	4	0.62 (0.14 to 1.11)^c^	79.22 (44.53 to 92.21)
	Long-term effect	2	0.27 (0.02 to 0.53)^c^	0.00
**Positive affect**				
	Short-term effect	6	0.19 (−0.04 to 0.42)	0.00 (0.00 to 74.63)
	Long-term effect	4	0.09 (−0.22 to 0.39)	0.00 (00.00 to 84.69)
**Negative affect**				
	Short-term effect	6	0.28 (0.05 to 0.51)^c^	0.00 (0.00 to 74.63)
	Long-term effect	4	0.16 (−0.15 to 0.46)	0.00 (00.00 to 84.69)

^a^Number of comparisons.

^b^*P*<.001.

^c^*P*<.05.

## Discussion

### Principal Findings

A total of 32 RCTs were found in our systematic review and meta-analysis to have evaluated the efficacy of CAIs in easing the symptoms of a range of mental health problems. With effect sizes ranging from *g*=0.24 to 0.62, most of which remained robust even after performing various sensitivity analyses, they were significantly better than control conditions in improving depressive symptoms, generalized anxiety symptoms, specific anxiety symptoms, quality of life or well-being, general distress, stress, mental disorder symptoms, psychosomatic disease symptoms, and negative affect. The long-term effects of CAIs on depressive symptoms, general distress, stress, mental disorder symptoms, and psychosomatic disease symptoms were statistically significant (*g*=0.16-0.39). More high-quality evidence is needed in the future to explore the long-term efficacy of CAIs.

Different mental health problems responded well to the 4 different types of CAs. Chatbot showed the largest effect size for generalized anxiety symptoms and quality of life, ECA showed the largest effect size for depressive symptoms and stress, CA in VR showed the largest effect size for specific anxiety symptoms, and avatar showed the largest effect size for general distress. This suggested that the efficacy of the different digital methods varied.

When compared with active controls, only the CAIs for depressive symptoms and general distress showed statistically significant effect sizes. Significant effect sizes were observed in the nonsymptomatic (depressive symptoms, generalized anxiety symptoms, quality of life, and general distress), symptomatic (depressive symptoms, generalized anxiety symptoms, and quality of life), and clinical samples (depressive symptoms, specific anxiety symptoms, and general distress).

Although there was no statistically significant correlation between the length of the intervention and its efficacy, we recommend extending it to better determine how clinical outcomes and nonclinical metrics interact during the intervention of CAIs.

CAs with advanced empathetic skills have improved user affinities and experiences [77,78]. In mental health CAs, the use of user profiles or user models to support personalized and adaptive features and the assessment of personalization is still in its infancy [79]. The results of this meta-analysis demonstrate how the use of personalization and empathic responses can significantly improve the efficacy of CAIs. In particular, empathic responses were linked to larger effect sizes for all mental health outcomes. This suggests that future technology and mechanism research on CAs may concentrate on these 2 capacities. A breakthrough in these 2 capacities will be necessary for CAs to function as competently as human therapists.

We included automatic reminders and theoretical orientation as moderators in the subgroup analyses, as in the earlier meta-analyses of smartphone interventions. In contrast to previous research [72,80], which concluded that studies offering engagement reminders were consistently associated with larger effect sizes, our study showed a stronger effect on depressive symptoms when the intervention did not include automatic reminders to engage. The same phenomenon was observed in the effect sizes of generalized anxiety symptoms, general distress, and stress, although it was not statistically significant. This may be because digital interventions such as internet-based CBT largely consisted of linear, structured psychotherapy modules [81,82], whereas more freedom for participants was offered in the context of CAIs [12], where interference and repetition would instead have an adverse effect on interest. We learned that the design of CAs needs to be succinct and empathic because, otherwise, the user’s interest will quickly wane, which is not conducive to the establishment of a stable working alliance between CAs and participants. Coupled with feedback for CAIs of participants in some studies, such as “process violations, repetitive content, misunderstanding, impersonality, not enough interactivity, and unnatural conversation,” we think that “maintaining engagement in the therapeutic process” will be the next area of focus for the development of CAIs for mental health problems rather than “attracting participation into the therapeutic process.”

As found in depressive symptoms and generalized anxiety symptoms, dosage is significantly positively correlated with the efficacy of CAIs so that a moderate increase in the frequency of interaction between CAs and participants is beneficial. Future CAs work to increase participants’ willingness to actively engage in conversation was supposed rather than only requiring them to accomplish a task in a passive manner.

### Limitations and Future Directions

There were certain limitations in this study that should be considered. First, a thorough search was conducted, prioritizing sensitivity over specificity because of the lack of standardized language in this field. Although some of the included studies treated a specific mental health issue as a secondary outcome or auxiliary outcome, the data were still extracted and used in the corresponding meta-analysis, and we used the broadest range of participants and comparators, which may have resulted in significant heterogeneity. Second, we chose the measurement instruments that most frequently corresponded to each of the mental health outcome to extract the data. We then used the standardized mean difference to remove the effect of dimensionality; however, it should be noted that different measurement instruments for the same mental health outcome are not exactly equivalent. As a result, if sufficient research has been conducted, it makes more sense to use a single measurement tool or to perform additional subgroup analysis for the measurement tool. Finally, in meta-regression, participant characteristics can only be included as covariates at the study or trial level for analysis, which may not accurately reflect the level of individual participants, leading to *aggregate bias* [83].

### Conclusions

In conclusion, our study found evidence for the efficacy of existing CAIs for mental health problems and offered the most thorough summary of their clinical traits and nonclinical metrics. When compared with various control settings and across diverse groups, CAIs significantly improved a variety of mental health outcomes in the short term, but their long-term impacts were less than ideal. The performance and efficacy of 4 different forms of CAs (chatbot, ECA, CA in VR, and avatar) varied for various mental health problems. The efficacy of CAIs was strongly connected to 2 key facilitators: personalization and empathic response. Efficacy was not improved by receiving too many automated reminders to participate in CAIs. We also found a positive dose-response association.

In the postpandemic and digital eras, CAIs are likely to play a significant role and contribute significantly to the new health transformation. We still require more high-quality publications, particularly evidence of direct contrasts with guided digital interventions or web-based interventions. It is intended that multidisciplinary collaboration and integration continue to advance until the divide between theoretical mechanisms and technological development is effectively eliminated. Nevertheless, it is crucial to understand that CAIs are still in their infancy and have a long way to go before they can be widely used in clinical practice and reach their full potential within existing models of mental health care.

## Appendix

Multimedia Appendix 1 The supplemental content of PRISMA (Preferred Reporting Items for Systematic Reviews and Meta-Analyses), study characteristics, risk of bias, meta-analysis, subgroup analyses, and meta-regression.

## Abbreviations

CA: conversational agent
CAI: conversational agent intervention
CBT: cognitive behavioral therapy
ECA: embodied conversational agent
PRISMA: Preferred Reporting Items for Systematic Reviews and Meta-Analyses
RCT: randomized controlled trial
VR: virtual reality
WL: waitlist

## Data Availability

Deidentified data generated or analyzed during this study are available from the corresponding author on reasonable request.
