# Supplementary material for: Conversational Agent Interventions for Mental Health Problems: Systematic Review and Meta-analysis of Randomized Controlled Trials
Source: J Med Internet Res. 2023 Apr 28;25:e43862. doi: 10.2196/43862 (PMC10182468; doi:10.2196/43862)
Supplement: Multimedia Appendix 1 [file jmir_v25i1e43862_app1.pdf]

Supplemental Content

Table S1. PRISMA statement and checklist 2

Table S2. Summary of characteristics of included studies 5

Figure S1. Risk of bias summary 22

Figure S2. Risk of bias graph 23

Table S3. Begg's test and Egger's test results of short- and long-term effect for each mental health outcome 23

Figure S3. Funnel plot for short-term effects of CAIs 24

Figure S4. Funnel plot for long-term effects of CAIs 25

Figure S5. Forest plot for the long-term effects of CAIs on depressive symptoms 26

Figure S6. Bubble diagram of the significant results of meta-regression 27

Figure S7. Forest plot for the long-term effects of CAIs on generalized anxiety symptoms 28

Figure S8. Forest plot for the short-term effects of CAIs on specific anxiety symptoms 29

Figure S9. Forest plot for the long-term effects of CAIs on specific anxiety symptoms 29

Figure S10. Subgroup analyses of efficacy of CAIs for specific anxiety symptoms 30

Figure S11. Forest plot for the short-term effects of CAIs on quality of life ... 31

Figure S12. Forest plot for the long-term effects of CAIs on quality of life 31

Figure S13. Subgroup analyses of efficacy of CAIs for quality of life 32

Figure S14. Forest plot for the short-term effects of CAIs on general distress 33

Figure S15. Forest plot for the long-term effects of CAIs on general distress 33

Figure S16. Subgroup analyses of efficacy of CAIs for general distress 34

Figure S17. Forest plot for the short-term effects of CAIs on stress 35

Figure S18. Forest plot for the long-term effects of CAIs on stress 35

Figure S19. Subgroup analyses of efficacy of CAIs for stress 36

Figure S20. Forest plot for the short-term effects of CAIs on other outcomes 37

Figure S21. Forest plot for the long-term effects of CAIs on other outcomes 38

Table S1. PRISMA statement and checklist

Section and Topic	Item #	Checklist item	Location where item is reported
**TITLE**			
Title	1	Identify the report as a systematic review.	Cover page
**ABSTRACT**			
Abstract	2	See the PRISMA 2020 for Abstracts checklist.	Abstract
**INTRODUCTION**			
Rationale	3	Describe the rationale for the review in the context of existing knowledge.	Introduction
Objectives	4	Provide an explicit statement of the objective(s) or question(s) the review addresses.	Introduction
**METHODS**			
Eligibility criteria	5	Specify the inclusion and exclusion criteria for the review and how studies were grouped for the syntheses.	Methods
Information sources	6	Specify all databases, registers, websites, organisations, reference lists and other sources searched or consulted to identify studies. Specify the date when each source was last searched or consulted.	Methods
Search strategy	7	Present the full search strategies for all databases, registers and websites, including any filters and limits used.	Methods
Selection process	8	Specify the methods used to decide whether a study met the inclusion criteria of the review, including how many reviewers screened each record and each report retrieved, whether they worked independently, and if applicable, details of automation tools used in the process.	Methods
Data collection process	9	Specify the methods used to collect data from reports, including how many reviewers collected data from each report, whether they worked independently, any processes for obtaining or confirming data from study investigators, and if applicable, details of automation tools used in the process.	Methods
Data items	10a	List and define all outcomes for which data were sought. Specify whether all results that were compatible with each outcome domain in each study were sought (e.g. for all measures, time points, analyses), and if not, the methods used to decide which results to collect.	Methods
	10b	List and define all other variables for which data were sought (e.g. participant and intervention characteristics, funding sources). Describe any assumptions made about any missing or unclear information.	Methods
Study risk of bias assessment	11	Specify the methods used to assess risk of bias in the included studies, including details of the tool(s) used, how many reviewers assessed each study and whether they worked independently, and if applicable, details of automation tools used in the process.	Methods
Effect measures	12	Specify for each outcome the effect measure(s) (e.g. risk ratio, mean difference) used in the synthesis or presentation of results.	Methods
Synthesis methods	13a	Describe the processes used to decide which studies were eligible for each synthesis (e.g. tabulating the study intervention characteristics and comparing against the planned groups for each synthesis (item #5)).	Methods

Section and Topic	Item #	Checklist item	Location where item is reported
	13b	Describe any methods required to prepare the data for presentation or synthesis, such as handling of missing summary statistics, or data conversions.	Methods
	13c	Describe any methods used to tabulate or visually display results of individual studies and syntheses.	Methods
	13d	Describe any methods used to synthesize results and provide a rationale for the choice(s). If meta-analysis was performed, describe the model(s), method(s) to identify the presence and extent of statistical heterogeneity, and software package(s) used.	Methods
	13e	Describe any methods used to explore possible causes of heterogeneity among study results (e.g. subgroup analysis, meta-regression).	Methods
	13f	Describe any sensitivity analyses conducted to assess robustness of the synthesized results.	Methods
Reporting bias assessment	14	Describe any methods used to assess risk of bias due to missing results in a synthesis (arising from reporting biases).	Methods
Certainty assessment	15	Describe any methods used to assess certainty (or confidence) in the body of evidence for an outcome.	Methods
**RESULTS**			
Study selection	16a	Describe the results of the search and selection process, from the number of records identified in the search to the number of studies included in the review, ideally using a flow diagram.	Results
	16b	Cite studies that might appear to meet the inclusion criteria, but which were excluded, and explain why they were excluded.	Results
Study characteristics	17	Cite each included study and present its characteristics.	Results
Risk of bias in studies	18	Present assessments of risk of bias for each included study.	Results
Results of individual studies	19	For all outcomes, present, for each study: (a) summary statistics for each group (where appropriate) and (b) an effect estimate and its precision (e.g. confidence/credible interval), ideally using structured tables or plots.	Results
Results of syntheses	20a	For each synthesis, briefly summarise the characteristics and risk of bias among contributing studies.	Results
	20b	Present results of all statistical syntheses conducted. If meta-analysis was done, present for each the summary estimate and its precision (e.g. confidence/credible interval) and measures of statistical heterogeneity. If comparing groups, describe the direction of the effect.	Results
	20c	Present results of all investigations of possible causes of heterogeneity among study results.	Results
	20d	Present results of all sensitivity analyses conducted to assess the robustness of the synthesized results.	Results
Reporting	21	Present assessments of risk of bias due to missing results (arising from reporting biases) for each synthesis assessed.	Results

Section and Topic	Item #	Checklist item	Location where item is reported
biases			
Certainty of evidence	22	Present assessments of certainty (or confidence) in the body of evidence for each outcome assessed.	Results
**DISCUSSION**			
Discussion	23a	Provide a general interpretation of the results in the context of other evidence.	Discussion
	23b	Discuss any limitations of the evidence included in the review.	Discussion
	23c	Discuss any limitations of the review processes used.	Discussion
	23d	Discuss implications of the results for practice, policy, and future research.	Discussion
**OTHER INFORMATION**			
Registration and protocol	24a	Provide registration information for the review, including register name and registration number, or state that the review was not registered.	Methods
	24b	Indicate where the review protocol can be accessed, or state that a protocol was not prepared.	Methods
	24c	Describe and explain any amendments to information provided at registration or in the protocol.	Methods
Support	25	Describe sources of financial or non-financial support for the review, and the role of the funders or sponsors in the review.	Acknowledgements
Competing interests	26	Declare any competing interests of review authors.	Conflicts of Interest
Availability of data, code and other materials	27	Report which of the following are publicly available and where they can be found: template data collection forms; data extracted from included studies; data used for all analyses; analytic code; any other materials used in the review.	Data availability

Table S2. Summary of characteristics of included studies

Study	Participants	Intervention	Control	Intervention length	Follow-up length	Theory	Reminders to engage	Mental health outcomes	Engagement	Acceptability and user experience
Fitzpatrick et al, 2017 (United States)	Symptomatic sample from US university, recruited on social media; mean age 22.19 years (SD 2.33); 67% (47/70) female	Woebot (n=34), classified as chatbot	ebook (n=36), classified as information control	2-3 weeks	No follow-up	CBT	Periodical automated reminder	Depression (PHQ-9), Anxiety (GAD-7), Positive affect (PANAS), Negative affect (PANAS)	17% (12/70) attrition, 9% (3/34) in intervention, 31% (11/36) in control; mean frequency of interaction 12.14 times (SD 2.23) over the 2 weeks period	Participants in the Woebot condition scored higher on overall satisfaction (*p*<.001), content satisfaction (*p*=.020) and emotional awareness (*p*=.021) compared with control condition; Participants' best experiences were accountability (n=9), empathy (n=7) and conversation (n=3); Participants' worst experiences were process violations (n=15), technical problems (n=8) and problems with content (n=8)
Fulmer et al, 2018 (United States)	Symptomatic sample from US university, recruited on social media; mean age 22.90 years (SD 4.08); 69% (52/75) female	Tess (group 1, n=24), Tess (group 2, n=26), classified as chatbot	ebook (n=25), classified as information control	2-4 weeks	No follow-up	CBT	Periodical automated reminder	Depression (PHQ-9), Anxiety (GAD-7), Positive affect (PANAS), Negative affect (PANAS)	1% (1/75) attrition (control group); mean interaction 283 messages (SD 147.6) in group 1 over the 2 weeks period, 286 messages (SD 104.6) with group 2 over the 4 weeks period	In the Tess condition, 86% (43/50) participants were overall satisfied with Tess, 80% (40/50) were satisfied with content of Tess, 86% (43/50) extended emotional awareness, 80% (40/50) learned something new from Tess, 80% (40/50) obtained information relevant to everyday life, 64% (32/50) felt more comfortable with therapeutic process; Participants' best

										experiences were accessibility (n=14), empathy (n=6) and learning (n=11); Participants' worst experiences were limitations in natural conversation (n=12), misunderstanding (n=11) and not enough interactivity (n=7)
Greer et al, 2019 (United States)	Symptomatic sample from young people after cancer treatment, recruited on social media; mean age 25 years (SD 2.9; range: 19-29); 80% (36/45) female	Vivibot (n=25), classified as chatbot	WL (n=20), classified as passive control	4 weeks	4 weeks	PCT	Periodical automated reminder	Depression (PROMIS), Anxiety (PROMIS), Positive affect (DES), Negative affect (DES)	27% (12/45) attrition at 2 weeks, 32% (8/25) in intervention, 20% (4/20) in control; 27% (12/45) attrition at 4 weeks, 36% (9/25) in intervention, 15% (3/20) in control; 42% (19/45) attrition at 8 weeks, 56% (14/25) in intervention, 25% (5/20) in control; mean interaction 12.1 times (SD 7.1) and 73.8 minutes (SD 52) over the 4 weeks period	Participants in the Vivibot condition felt helpful chatting with Vivibot, with an average rating of 2.03 (SD 0.72; range: 0-3), and they were likely to recommend Vivibot to a friend, with an average rating of 6.9 (SD 2.6; range: 0-10). When asked why they were likely or unlikely to recommend Vivibot to a friend, participants remarked on the utility and nonjudgmental nature of talking to an automated agent
Jang et al,	Clinical	Todaki	Paperback	4 weeks	No follow-up	CBT	No	ADHD	20% (9/46)	There was no significant

2021 (Korea)	sample from psychiatry clinic in Gangnam Severance Hospital, recruited with posters; mean age 24.79 years (SD 7.34); 57% (26/46) female	(n=23), classified as chatbot	(n=23), classified as information control				reminder	(CAARS), Depression (QIDS-SR), Anxiety (SAS), Stress (PSS Korean version)	attrition, 17% (4/23) in intervention, 22% (5/23) in control; mean interaction 20.32 times (SD 12.89) and 75 minutes (SD 80) over the 4 weeks period	difference between the Todaki and control group on overall satisfaction, content satisfaction, increased attention, new things learning, information relevant to daily life and therapeutic process satisfaction; Participants' best experiences were empathic/friendly mascot (n=7), daily check-up (n=6) and easy explanation (n=6); Participants' worst experiences were unnatural conversation (n=7) and non-intuitive interfaces (n=6)
Oh et al, 2020 (Korea)	Clinical sample from psychiatry clinic in Gangnam Severance Hospital, recruited with posters; mean age 40.97 years (SD 11.4); 51% (21/41) female	Todaki (n=23), classified as chatbot	Paperback (n=22), classified as information control	4 weeks	No follow-up	CBT	Periodical automated reminder	Panic disorder (PDSS, APPQ), Depression (HADS), Anxiety (HADS)	9% (4/45) attrition, 9% (2/23) in intervention, 9% (2/22) in control; mean interaction 9 days (SD 6.7) and 50 minutes (SD 34.6) over the 4 weeks period	There were no significant differences in SUS between the Todaki (64.5±17.0) and control group (69.5±17.2); Participants' best experiences were reliability (n=8), ease interface (n=9) interactive learning (n=7) and coping training (n=9); Participants' worst experiences were repetitive pattern (n=7), familiarity with the learning contents (n=7), lack of diversity (n=9) and wrong answers (n=7)
Bennion et al, 2020 (United	General sample from the	MYLO (n=59), classified as	ELIZA (n=53), classified as	>20 minutes	2 weeks	MOL	No reminder	Problem solving (Problem	13% (14/112) attrition at post-intervention,	Participants in the MYLO condition scored higher on helpfulness (*p*=.003),

Kingdom)	University of the Third Age (U3A), recruited on U3A websites and U3A meeting places; mean age 69.21 years (SD 6.76; range: 51-90); 73% (82/112) female	chatbot	active control					distress scale, Problem resolution scale), Depression (DASS-21), Anxiety (DASS-21), Stress (DASS-21)	19% (11/59) in intervention, 6% (3/53) in control; 16% (18/112) attrition at 4 weeks, 20% (12/59) in intervention, 11% (6/53) in control; mean interaction 24.17 minutes (SD 16.46) over one session	tendency of using again (*p*=.004) and tendency of using the conversational agent for a future problem (*p*=.005) compared with control condition; There were no significant differences in SUS between MYLO (63.56±17.90) and ELIZA (56.97±19.46); linear regression with the MYLO system usability as the predictor variable revealed a significant relationship between the MYLO system usability and helpfulness ($P<.001$; R^2=0.299), use again ($P<.001$; R^2=0.294) and problem resolution (P=.003; R^2=0.176)
Bird et al, 2018 (United Kingdom)	Symptomatic sample from the University of Manchester and the University of Liverpool, recruited via websites and with posters; mean age 22.08 years (SD 7.19;	MYLO (n=85), classified as chatbot	ELIZA (n=86), classified as active control	>15 minutes	2 weeks	MOL	No reminder	Problem solving (Problem distress scale, Problem resolution scale), Depression (DASS-21), Anxiety (DASS-21), Stress (DASS-21)	No attrition at post-intervention; 61% (104/171) attrition at 4 weeks, 62% (53/85) in intervention, 59% (51/86) in control; mean interaction 13 minutes over one session	No assessment

	range: 16-70); 82% (160/196) female									
Gaffney et al, 2014 (United Kingdom)	Symptomatic sample from the University of Manchester, recruited via posters and websites; mean age 21.4 years (SD 3.1; range: 18-32); 79% (33/42) female	MYLO (n=26), classified as chatbot	ELIZA (n=22), classified as active control	20 minutes	2 weeks	MOL	No reminder	Problem solving (Problem distress scale), Depression (DASS-21), Anxiety (DASS-21), Stress (DASS-21)	10% (5/48) attrition at post-intervention, 15% (4/26) in intervention, 5% (1/22) in control; 13% (6/48) attrition at 2 weeks, 15% (4/26) in intervention, 9% (2/22) in control; mean interaction 19.23 minutes (SD 0.002) over one session	Participants in the MYLO condition scored higher on helpfulness (*p*<.05) compared with control condition; mediation analysis indicated that participants who have greater positive expectancies and lower negative expectancies towards computer-based problem solving experienced significantly larger problem resolution
Ly et al, 2017 (Sweden)	General sample from Swedish universities, recruited on social media; mean age 26.2 years (SD 7.2; range: 20-49); 54% (15/28) female	Shim (n=14), classified as chatbot	WL (n=14), classified as passive control	2 weeks	No follow-up	CBT	No reminder	Well-being (FS, SWLS), Stress (PSS-10)	No attrition; mean interaction 8.21 days (SD 3.0) and 17.71 times (SD 15.7) over the 2 weeks period	The themes of user experience were content with the subthemes activation (n=5), learning (n=2), reflection (n=3), repetitiveness (n=5) and shallowness (n=3); medium with the subthemes routine (n=4), moderator (n=3), availability (n=3) and lack of clarity (n=1); and functionalities with the subthemes weekly summary (n=2), lack of notifications (n=1) and restricted UI (n=1)

Prochaska et al, 2021 (United States)	Symptomatic sample from adults with substance use concerns, recruited on social media; mean age 40 years (SD 12; range: 20-49); 65% (117/180) female	W-SUDs (n=88), classified as chatbot	WL (n=92), classified as passive control	8 weeks	No follow-up	CBT	Periodical automated reminder	SUD (SUO, Pain rating), Depression (PHQ-8), Anxiety (GAD-7)	16% (28/180) attrition, 19% (17/88) in intervention, 12% (11/92) in control; mean interaction 34.9 days (SD 28.9) and 1052 messages (SD 878) over the 8 weeks period	Participants in the W-SUDs condition got an average rating of 29.0 (SD 4.8; range: 6-36) on URP-I-Acceptability, 32.0 (SD 3.6; range: 6-36) on URP-I- Feasibility, 25.5 (SD 5.0; range: 8-32) on CSQ-8, 14.4 (SD 4.7; range: 4-20) on WAISR-Goal, 13.9 (SD 4.5; range: 4-20) on WAISR-Task and 15.7 (SD 4.6; range: 4-20) on WAISR-Bond.
Hauser-Ulrich et al, 2020 (Switzerland)	Symptomatic sample from the German-speaking part of Switzerland, recruited on social media; mean age 43.77 years (SD 12.72; range: 20-49); 80% (82/102) female	SELMA (n=59), classified as chatbot	WL (n=43), classified as passive control	8 weeks	No follow-up	CBT	Periodical automated reminder	Chronic pain (BPI, DSF), Well-being (MFHW)	40% (41/102) attrition, 36% (21/59) in intervention, 47% (20/43) in control; mean interaction with SELMA 200 conversations (SD 58.45) over the 8 weeks period	Participants in the SELMA condition scored higher on WAISR-Bond (*p*=.005) compared with control condition, 24/38 (63%) of the participants had fun using the app, 18/38 (47%) felt useful, and 31/38 (84%) agreed that it was easy to use. The duration of the program was exactly right for 19/38 (50%) of the participants. The number of messages was too short for 28/38 (74%) and their content was not sufficiently profound for 29/38 (76%) of the participants

Lavelle et al, 2021 (Ireland)	General sample from Ireland university, recruited on university study pools and social media; mean age 28.01 years (SD 10.29; range: 18-68); 78% (174/223) female	Littlebot (n=72), Little Chatbot (n=80), classified as chatbot	WL (n=71), classified as passive control	5 days	No follow-up	ACT	No reminder	Negative self-referential thoughts (The Target Thought Measure), Positive affect (PANAS), Negative affect (PANAS)	72% (161/223) attrition, 68% (49/72) in defusion group, 74% (49/80) in restructuring group, 75% (53/71) in control; no significant differences between the defusion and restructuring groups on adherence to intervention (*p* =.6)	No assessment
Maeda et al, 2020 (Japan)	General sample from women hoping to have children now or in the future, recruited via an online social research panel; mean age 28.8 years (SD 3.6); 100% (927/927)	Educational chatbot for fertility and preconcepti on health (n=309), classified as chatbot	PDF document (n=309), PDF document (n=309), classified as information control	1 hour	No follow-up	RLP	No reminder	Anxiety (STAI-S)	A total of 574 chatbot sessions were recorded, which had an average length of 8 minutes	Feedback from participants was technical problems (n=28), low comprehension (n=77), slow chatbot operation (n=15), quick information display (n=14), pros of using the chatbot (n=96), cons of using the chatbot (n=33), understanding promotion (n=28), burdensome and necessity (n=15), lack of humanity or empathy (n=17), appreciation for increased knowledge and awareness (n=114), superficial content or lack of details (n=30)

	female									
Hunt et al, 2021 (United States)	Symptomatic sample recruited on IBS-specific social media; mean age 32 years (SD 10.2; range: 18-63); 75% (91/121) female	Zemedy (n=62), classified as chatbot	WL (n=59), classified as passive control	8 weeks	3 months	CBT	Periodical automated reminder	IBS (GSRS-IBS), Depression (DASS-21), Anxiety (DASS-21), Quality of life (IBS-QOL), Stress (DASS-21)	51% (62/121) attrition at 8 weeks, 42% (26/62) in intervention, 61% (36/59) in control; 63% (76/121) attrition at 3 months, 61% (38/62) in intervention, 64% (38/59) in control; mean interaction with Zemedy 3.2 modules (SD 2) over the 8 weeks period	No assessment
Klos et al, 2021 (Argentina)	General sample from college students in Entre Ríos, recruited via presentations in university courses, aged 18-33 years; 87% (158/181) female	Tess (n=99), classified as chatbot	ebook (n=82), classified as information control	8 weeks	No follow-up	CBT	Periodical automated reminder	Anxiety (GAD-7)	60% (108/181) attrition, 39% (39/99) in intervention, 41% (34/82) in control; mean interaction with Tess 472 messages (SD 249.52; range 162-1290) over the 8 weeks period	Feedback from participants (coded as positive, negative, or ambivalent) was associated with the number of messages exchanged with Tess
Liu et al, 2022	Symptomatic sample	XiaoNan (n=41),	ebook (n=42),	4 weeks /8 weeks /12	No follow-up	CBT	No reminder	Depression (PHQ-9),	24% (20/83) attrition at 16	Participants in the XiaoNan condition scored higher on

(China)	from China universities, recruited with online poster; mean age 23.08 years (SD 1.76; range: 19-28); 55% (46/83) female	classified as chatbot	classified as information control	weeks /16 weeks				Anxiety (GAD-7), Positive affect (PANAS), Negative affect (PANAS)	weeks, 20% (8/41) in intervention, 29% (12/42) in control	WAI-SR (*P*<.01) compared with control, whereas no significant differences on the CSQ-8 scores (*P*=0.38). There were no significant differences between the self-reported adherence rates of the two groups (*P*=0.50); Participants' best experiences were accessibility (n=11), empathy/Friendly (n=8) and depression exploration (n=9); Participants' worst experiences were impersonality (n=8), repetitive contents (n=10) and overgeneralization (n=8)
Ali et al, 2020 (United States)	Clinical sample from community and an outpatient geriatric psychiatry clinic; mean age 71.47 years (SD 7.51); 68% (13/19) female	APE (n=10), classified as ECA	Online materials (n=9), classified as information control	4-6 weeks	No follow-up	CBT	Periodical automated reminder	Communication difficulty (SSPA)	5% (1/19) attrition (AEP group)	The system usability score for the AEP was 69.5
Burton et al, 2016 (United	Clinical sample from Romania,	Help4Mood+ TAU (n=14), classified as	TAU (n=14), classified as active	4 weeks	No follow-up	CBT	Periodical automated reminder	Depression (BDI-2, QIDS-SR,	25% (7/28) attrition, 14% (2/14) in	Feedback from participants was ability to customise gender and appearance,

Kingdom)	Spain and UK, recruited via invitation in writing by clinicians; mean age 38.77 years (SD 11.03); 67% (18/27) female	ECA	control					DAS-SF2), Quality of life (EQ-5D-5L),	intervention, 36% (5/14) in control; mean interaction with Help4Mood 10.5 times and 134 minutes over the 4 weeks period	responsiveness and relationship, too cold and repetitive content, realism and the opportunity to tailor session length to their needs
Suganuma et al, 2018 (Japan)	General sample from company employees, university students and housewives, recruited via an internet research company; mean age 38.05 years (SD 12.37); 70% (318/454) female	SABORI (n=559), classified as ECA	WL (n=2109), classified as passive control	1 month	No follow-up	CBT	No reminder	Well-Being (WHO-5-J), Psychological distress (K10 Japanese Version)	83% (2214/2668) attrition, 66% (368/559) in intervention, 88% (1846/2109) in control; mean interaction with SABORI 10.5 times and 134 minutes over the 4 weeks period	No assessment
Loveys et al, 2021 (New Zealand)	Symptomatic sample from older adult recruited in 5 retirement	Bella (n=15), classified as ECA	WL (n=15), classified as passive control	1 week	2 weeks	HCI	Periodical automated reminder	Well-Being (FS), Loneliness (UCLA Loneliness Scale),	23% (7/30) attrition, 27% (4/15) in intervention, 20% (3/15) in control; mean	Participants reported that Bella was helpful for promoting resilience and well-being, with an average rating of 4.39 (SD 1.83; range: 0-7), and for

	village sites, and young adult recruited via email, social media , word of mouth and presentation ; mean age 68.20 years (SD 18.95); 80% (24/30) female							Distress (COVID-19 Distress Scale), Stress (PSS-4), Positive affect (SPANE), Negative affect (SPANE)	interaction with Bella 6.23 times (SD 1.19) and 20.20 minutes (SD 13.95) over the 7 days period	improving any feelings of loneliness, with an average rating of 4.09 (SD 1.76; range: 0-7); Participants were willing to use Bella again in the future, with an average rating of 4.09 (SD 1.98; range: 0-7); Participants' best experiences were appearance, speech, interpersonal skills, informational support, user experience and novel technology; Participants' worst experiences were interaction behaviors, conversation design, robotic speech, technology advances, preference for a real human and no changes
Sandoval et al, 2017 (United States)	Symptomatic sample from adult with major depressive disorder or dysthymia, recruited from the local media, university health centers, hospitals,	imbPST (n=25), classified as ECA	WL (n=20), classified as passive control	6 weeks	No follow-up	PST	No reminder	Depression (BDI-2, HSCL-20-d)	No attrition	The SUS score of participants in the imbPST condition improved significantly over 6 weeks (F=20.7, p<.001)

	churches, workplaces and community center; mean age 28.78 years (SD 9.13); 62% (28/45) female									
Cartreine et al, 2012 (United States)	Symptomatic sample from persons who bear some demographic similarities to astronauts, recruited via email; mean age 50.35 years (SD 8.68); 71% (10/14) female.	ePST (n=7), classified as ECA	WL (n=7) , classified as passive control	4 weeks	No follow-up	PST	No reminder	Depression (HDI)	21% (3/14) attrition, 14% (1/7) in intervention, 29% (2/7) in control; mean interaction with ePST 53 (SD 31) minutes over the 4 weeks period	Participants in the ePST condition had no significant difference on the SUS score between baseline and post-intervention (P<.09; Wilcoxon signed rank test).
Freeman et al, 2018 (United Kingdom)	Symptomatic sample from people with a fear of heights, recruited on local radio; mean age 45.5 years	Now I Can Do Heights (n=49), classified as CA in VR	WL (n=51), classified as passive control	2 weeks	4 weeks	CBT	No reminder	Acrophobia (HIQ, AQ)	No attrition; mean interaction with Now I Can Do Heights 4.66 sessions (SD 1.27) and 124.43 minutes (SD 34.23) over the 4 weeks	Mean increase of discomfort level (as assessed by the SSQ) 2.21 (95% CI 1.24-3.18; *P*<.0001) after entering VR for the first time; Mean increase of discomfort level (as assessed by the SSQ) 1.35 (95% CI 0.55-2.16; *P* =.002) after entering the last

	(range: 30-53); 52% (52/100) female								period	session of VR
Miloff et al, 2019 (Sweden)	Symptomatic sample from people with spider phobia, recruited on online and print media; mean age 34.05 years (SD 10.35); 83% (83/100) female	VIMSE (n=50), classified as CA in VR	In-vivo one-session treatment (n=50), classified as active control	3 hours	3 months /12 months	VRET	No reminder	Spider phobia (BAT, FSQ, SPQ), Depression (PHQ-9), Anxiety (GAD-7), Quality of life (BBQ)	3% (3/100) attrition at post-intervention, 4% (2/50) in intervention, 2% (1/50) in control; 11% (11/100) attrition at 3 months, 10% (5/50) in intervention, 12% (6/50) in control; 9% (9/100) attrition at 12 months, 6% (3/50) in intervention, 12% (6/50) in control	No significant difference between OST and VRET on the NEQ-32 (p=.145); The most highly reported negative effects of VRET participants were unfulfilled expectations for the treatment (n=19), treatment with no results produced (n=18) and ill-suited treatment (n=15). Experience of being present in a virtual environment was measured by IPQ, and no significant differences were observed for those with high-presence as compared to those with low-presence on BAT (*P*=.14), FSQ (*P*>.16) and SPQ (*P*>.16).
Bentz et al, 2021 (Switzerland)	Clinical sample from people with a fear of heights, recruited via print, radio and online advertisements; mean age 31.54	Easy Heights (n=39), classified as CA in VR	WL (n=38), classified as passive control	1 hour/3-5 weeks	No follow-up	VRET	No reminder	Acrophobia (BAT, ATHQ, AQ)	9% (7/77) attrition at post-phase 1, 13% (5/39) in intervention, 5% (2/38) in control; 39% (30/77) attrition at post-phase 2, 44% (17/39) in intervention,	No assessment

	years (SD 10.52); 49% (34/70) female								34% (13/38) in control;	
Donker et al, 2019 (Netherlands)	Symptomatic sample from the Dutch general population, recruited on websites, magazines, and local media; mean age 41.32 years (SD 13.64); 67% (129/193) female	ZeroPhobia (n=96), classified as CA in VR	WL (n=97), classified as passive control	3 weeks	3 months	CBT	Periodical automated reminder	Acrophobia (AQ, ATHQ), Depression (PHQ-9), Anxiety (BAI)	25% (49/193) attrition at 3 weeks, 41% (39/96) in intervention, 10% (10/97) in control; 31% (59/193) attrition at 3 months (intervention group)	The VR-CBT app participants scored an average of 42.69 (SD 10.40; range: 14-70) on IPQ. The VR-CBT app was rated as user friendly (mean [SD], 75.35 [14.74]). No deterioration or negative effects were identified measured by SSQ, except for 24 participants who reported 1 or more symptoms of transient cyber sickness.
Lindner et al, 2019 (Sweden)	General sample from the general public, recruited on television, newspaper and public bulletin boards at university campuses; mean age 31.36 years	Self-led OST+TAU (n=25), classified as CA in VR	Therapist-led OST+TAU (n=25), classified as active control	1 week	6 months /12 months	VRET	Periodical automated reminder	PSA (PSAS, LSAS, FNE), Depression (PHQ-9), Anxiety (GAD-7), Quality of life (BBQ)	10% (5/50) attrition at post-intervention, 16% (4/25) in intervention, 4% (1/25) in control; 16% (8/50) attrition at follow-up, 20% (5/25) in intervention, 12% (3/25) in control; mean exposure	Two groups scored similarly on CSQ-8 (*P*=.19) and NEQ-32 (P=.322)

	(SD 7.22); 72% (36/50) female								exercises with Self-led OST 3.28 times (SD 4.29) over the 7 days period	
Craig et al, 2018 (United Kingdom)	Clinical sample recruited via clinician; mean age 42.7 years (SD 10.7); 32% (48/150) female	AVATAR therapy (n=75), classified as avatar	TAU (n=75), classified as active control	12 weeks	24 weeks	/	No reminder	Depression (Calgary Depression Scale), Anxiety (DASS-21), Quality of life (MANSA), Stress (DASS-21)	17% (26/150) attrition at 12 weeks, 16% (12/75) in intervention, 19% (14/75) in control; 23% (35/150) attrition at 24 weeks, 24% (18/75) in intervention, 23% (17/75) in control; mean interaction 5.6 sessions (SD 2.8; range: 0-10) over the 12 weeks period	No assessment
Dellazizzo et al, 2021 (Canada)	Clinical sample recruited from the university and the community; mean age 42.5 years (SD 12.7); 24% (18/74) female	VR (n=37), classified as avatar	Cognitive–behavioral therapy (n=37), classified as active control	3 months-1 year	3 months /6 months /12 months	VRET	No reminder	Depression (PANSS, BDI-II), Anxiety (PANSS), Quality of life (QLESQ-SF)	16% (12/74) attrition at post-endpoint, 22% (8/37) in intervention, 11% (4/37) in control; 23% (17/74) attrition at 3 months, 27% (10/37) in intervention, 19% (7/37) in	In the 15 patients having participated in semi-structured interviews on their perspectives concerning treatments, most participants found their corresponding intervention to be adequate in content, sequence, dose, tailoring, timing, mode of delivery, and equipment use. One-third of participants did find the dose of interventions

									control; 55% (41/74) attrition at 6 months, 65% (24/37) in intervention, 46% (17/37) in control; 59% (44/74) attrition at 12 months, 65% (24/37) in intervention, 54% (20/37) in control	to be too short and would have preferred supplementary sessions. Particularly related to VRT, 37.5% voiced the intervention as being stressful at first, which is precisely within the scope of VRT.
Kocur et al, 2021 (Germany)	Clinical sample from inpatients with major depression at the university psychiatric hospital in Regensburg; mean age 37 years (SD 12.68; range: 20-59); 62% (21/34) female	CAT-DB+TAU (n=18), classified as avatar	TAU (n=16), classified as active control	5 days	14 days	CBT	No reminder	Depression (BDI-II, Conviction Rating)	No attrition at post-intervention; 24% (8/34) attrition at follow-up, 22% (4/18) in intervention, 25% (4/16) in control;	No assessment
Pinto et al, 2013 (United States)	General sample from young adults, recruited on	eSMART-MH (n=14), classified as avatar	Screen-based health education (n=14),	4 weeks/8 weeks	3 months	/	No reminder	Depression (HADS)	No attrition	No assessment

	communities; mean age 22 years (SD 2.2; range: 18-25); 64% (18/28) female		classified as attentional control							
du Sert et al, 2018 (Canada)	Symptomatic sample recruited from the university and the community; mean age 42.9 years (SD 12.4; range: 24-62); 33% (5/15) female	AVATAR therapy (n=15), classified as avatar	TAU (n=7), classified as active control	7 weeks	3 months	CBT	No reminder	Depression (BDI-II), Quality of life (QLESQ-SF)	No attrition	Participants rated their avatar credible enough (mean score of 7.5 on a scale of 10; SD = 1.5)

Abbreviation: CBT, cognitive behavioral therapy; PHQ, The Patient Health Questionnaire; GAD, The Generalized Anxiety Disorder; PANAS, The Positive and Negative Affect Schedule; PCT, patient centered therapy; PROMIS, Patient-Reported Outcomes Measurement Information System; DES, Differential Emotions Scale; CAARS, Conner s Adult ADHD Rating Scale; QIDS-SR, Quick Inventory of Depressive Symptomatology-Self-report; SAS, Self-rating Anxiety Scale; PSS, Perceived Stress Scale; PDSS, Panic Disorder Severity Scale; APPQ, Albany Panic and Phobia Questionnaire; HADS, Hospital Anxiety and Depression Scale; SUS, The system usability scale; MOL, method of levels therapy; DASS, The Depression, Anxiety, and Stress Scales; FS, The Flourishing Scale; SWLS, The Satisfaction With Life Scale; SUO, Substance Use Occasions; URP-I, Usage Rating Profile-Intervention; CSQ, Client Satisfaction Questionnaire; WAISR, Working Alliance Inventory-Short Revised; BPI, Brief Pain Inventory; DSF, German Pain Survey; MFHW, Marburger Screening for Habitual Well-being; ACT, acceptance and commitment therapy; RLP, reproductive life plan; STAI-S, State-Trait Anxiety Inventory- state-anxiety; GSRS-IBS, Gastrointestinal Symptom Rating Scale-irritable bowel syndrome; IBS-QOL, Irritable Bowel Syndrome-Quality of Life; SSPA, Social Skills Performance Assessment; BDI, Beck Depression Inventory; QIDS-SR, the Quick Inventory of Depressive Symptoms-Self-Report; DAS-SF, Dysfunctional Attitude Scale-Short Form; EQ-5D-5L, EuroQol-5 Dimension-5 Level; WHO-5-J, World Health Organization-Five Well-Being Index; K10, Kessler 10; HCI, human-computer interaction; SPANE, The Scale of Positive and Negative Experiences; PST, problem-solving treatment; HSCL-20-d, Hopkins Symptom Checklist 20-Item Depression Scale; HDI, Hamilton Depression Inventory; HIQ, the Heights Interpretation Questionnaire; AQ, the acrophobia questionnaire; SSQ, Simulator Sickness Questionnaire; VRET, virtual reality exposure therapy; BAT, Behavioral Approach Test; FSQ, The Fear of Spiders Questionnaire; SPQ, The Spider Phobia Questionnaire; BBQ, The Brunnsviken Brief Quality of Life Inventory; NEQ, The Negative Effects Questionnaire; IPQ, Igroup Presence Questionnaire; ATHQ, the Attitudes Towards Heights Questionnaire; MANSA, Manchester Short Assessment of Quality of Life; QLESQ-SF, Quality of Life Enjoyment and Satisfaction Questionnaire-Short From.

Figure S1. Risk of bias summary

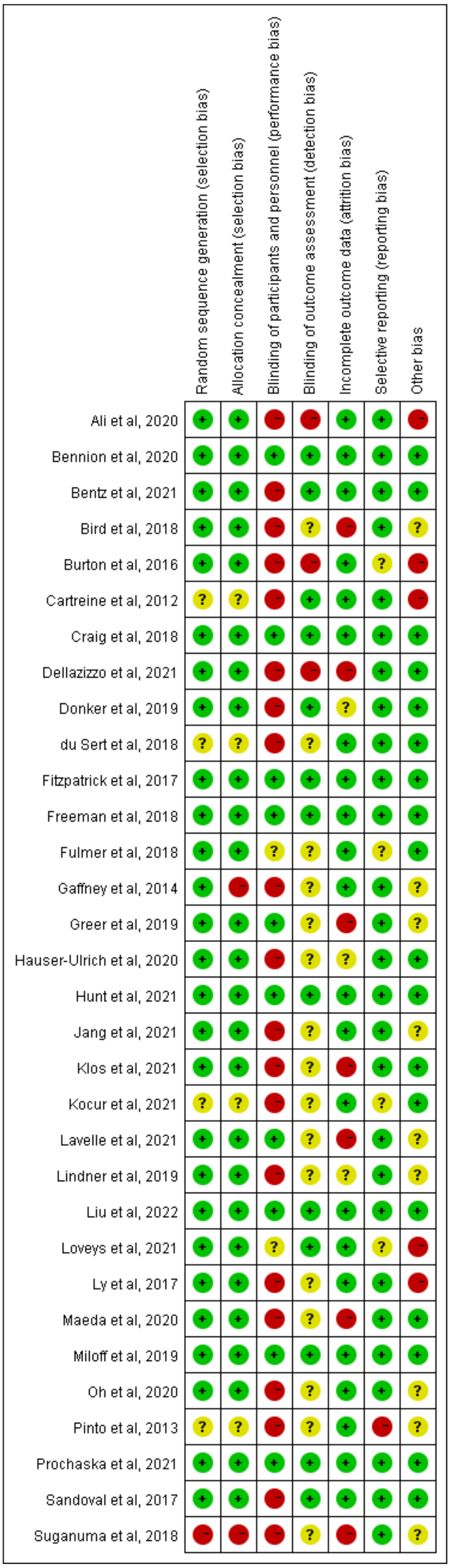

Figure S2. Risk of bias graph

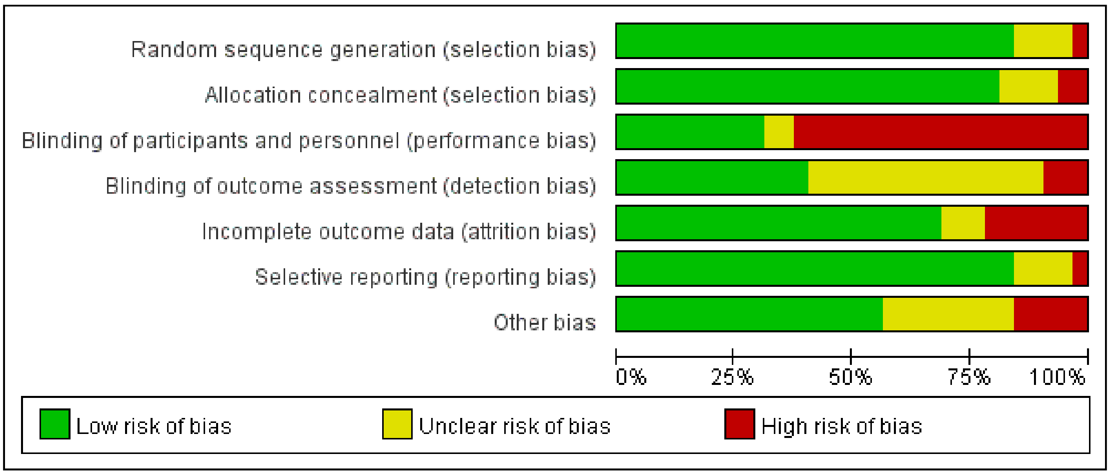

Among the included studies, 1 was assessed to have a high risk of bias due to poor randomization, 2 had a high risk of bias due to the lack of allocation concealment, 20 were assessed high on performance bias, 3 were assessed high on detection bias, 1 was assessed high on attrition bias, and 5 were assessed high on reporting bias. The interrater reliability suggested substantial agreement between the raters on each criterion of the Cochrane Collaboration's tool (Cohen's kappa = 0.86, 0.79, 0.67, 0.89, 0.93, 0.75 and 0.83, respectively).

Table S3. Begg's test and Egger's test results of short- and long-term effect for each mental health outcome

	**Short-term effect**		**Long-term effect**	
	Begg's test	Egger's test	Begg's test	Egger's test
Depressive symptoms	.055	.054	.463	.500
Generalized anxiety symptoms	.820	.356	.166	.119
Specific anxiety symptoms	.405	.422	.584	.624
Quality of life	.244	.585	.602	.994
General distress	.373	.090	.230	.487
Stress	.230	.332	.308	.070
Mental disorder symptoms	1.000	.393	1.000	.228
Psychosomatic disease symptoms	.734	.110	1.000	
Positive affect	.260	.108	.308	.540
Negative affect	.260	.363	.089	**.041**

Bold prints indicate significant differences.

Figure S3. Funnel plot for short-term effects of CAIs

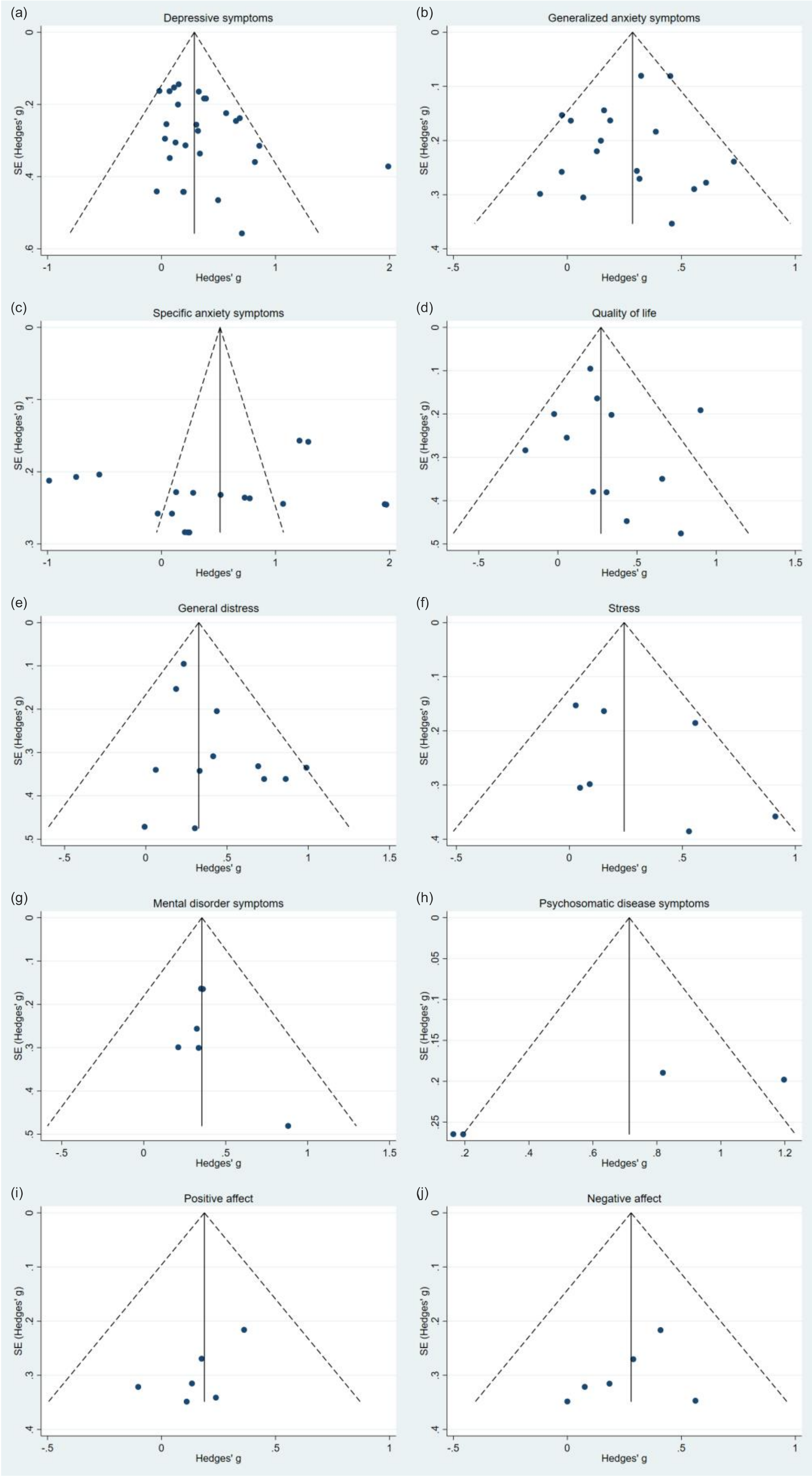

Figure S4. Funnel plot for long-term effects of CAIs

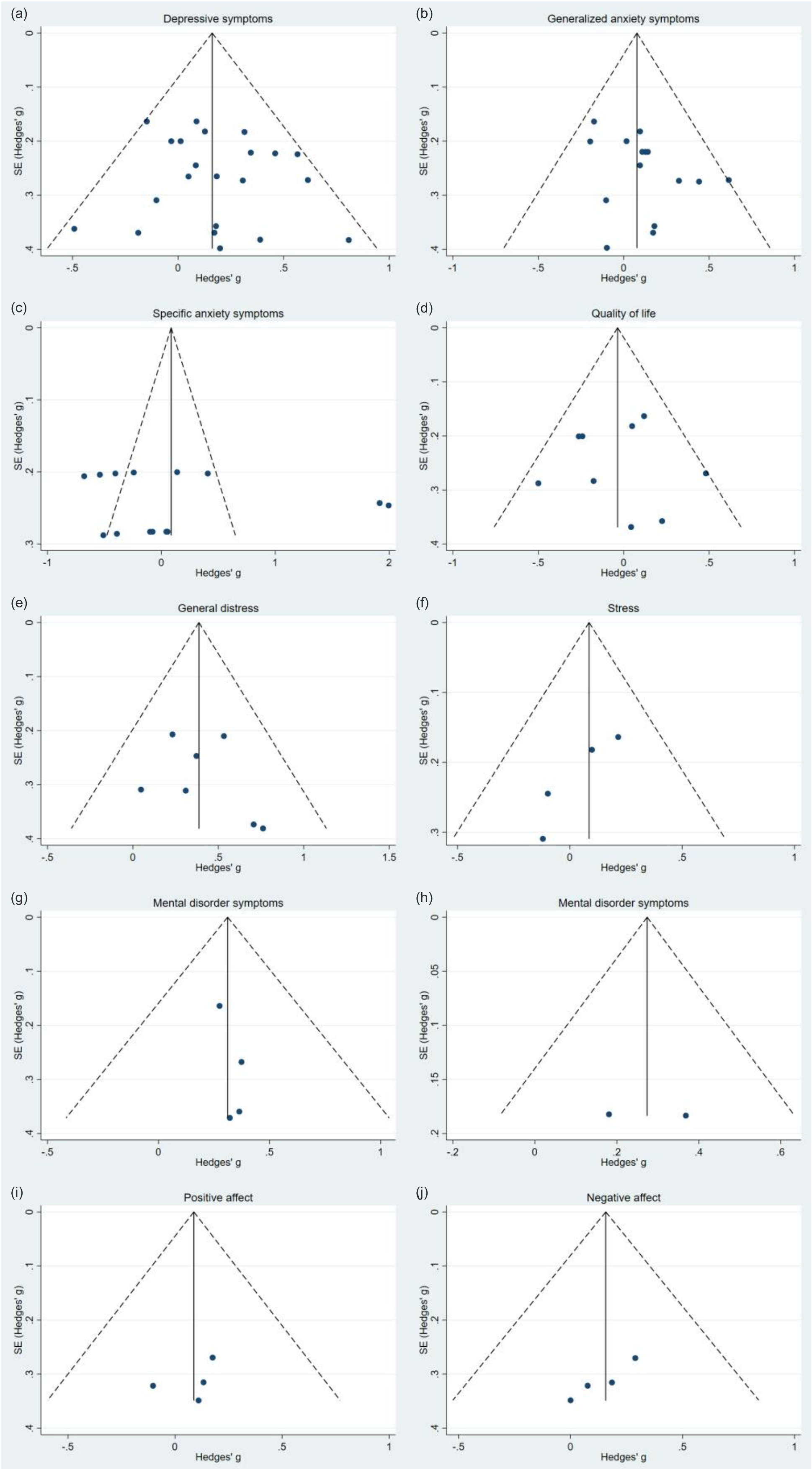

Figure S5. Forest plot for the long-term effects of CAIs on depressive symptoms

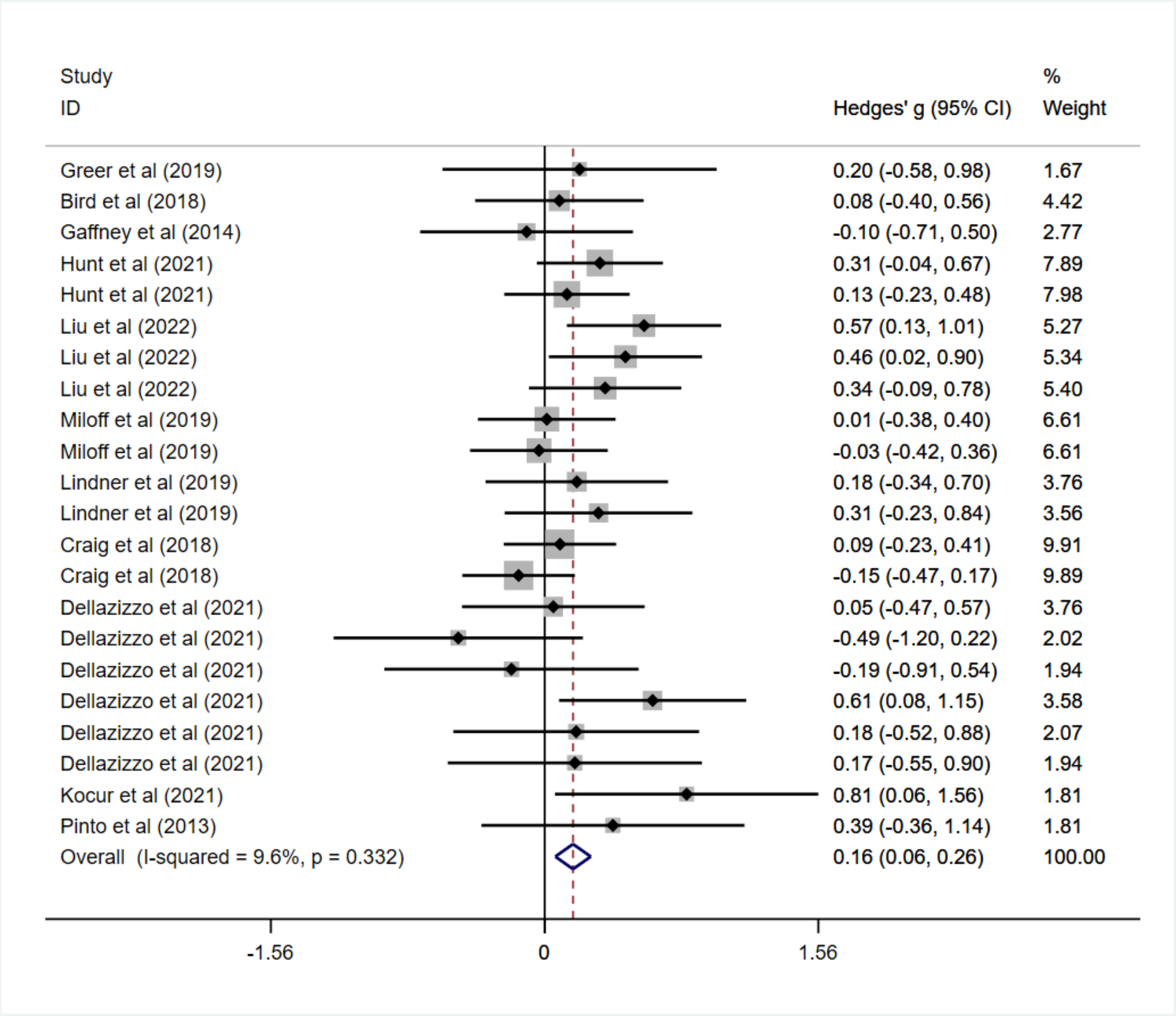

Figure S6. Bubble diagram of the significant results of meta-regression

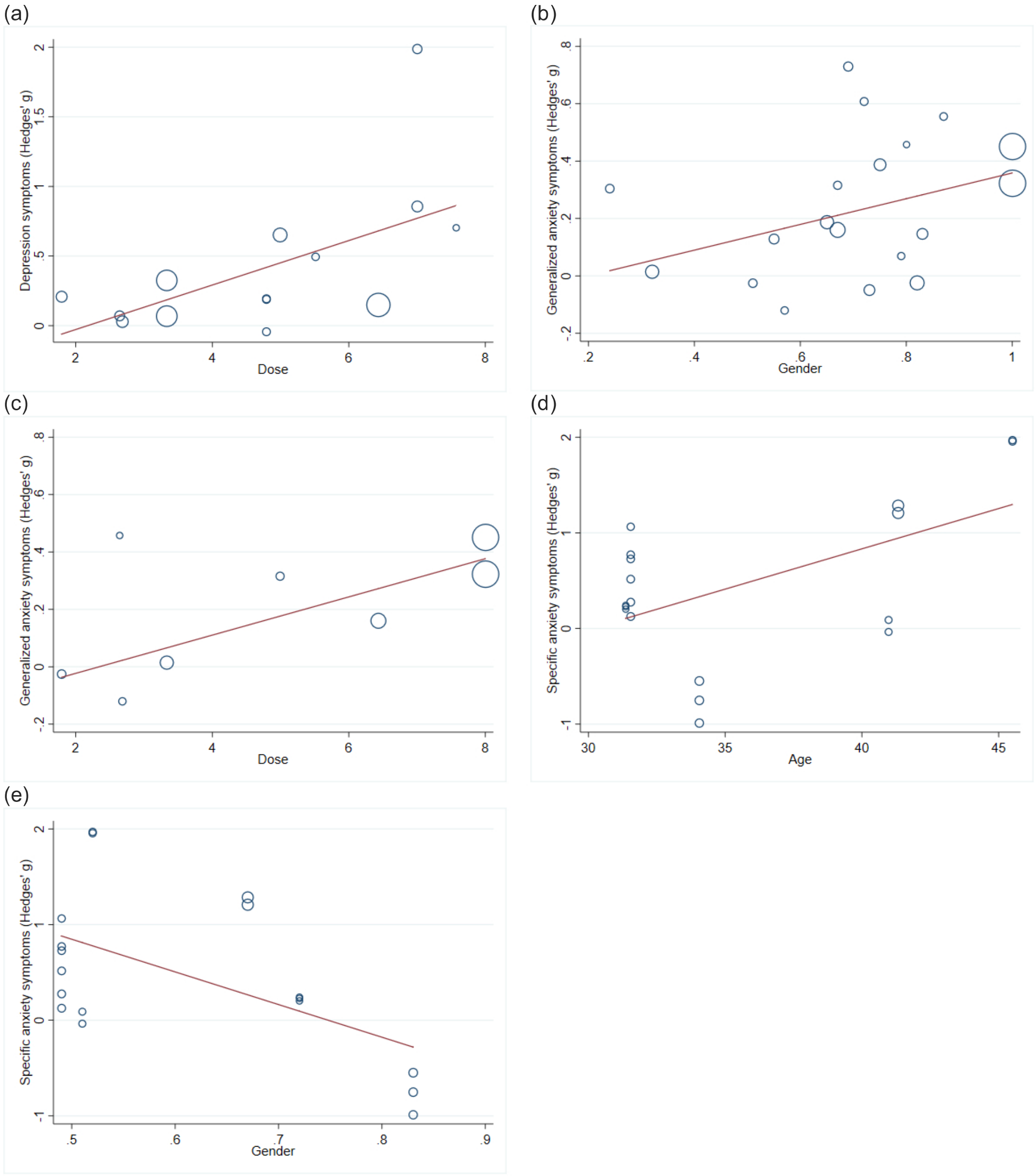

The figure shows the direction of effect size for mental health outcomes as a function of age, gender and dose. Circles are proportionate to study weight in the analysis. Dose, average duration of interaction with conversational agent per day.

Figure S7. Forest plot for the long-term effects of CAIs on generalized anxiety symptoms

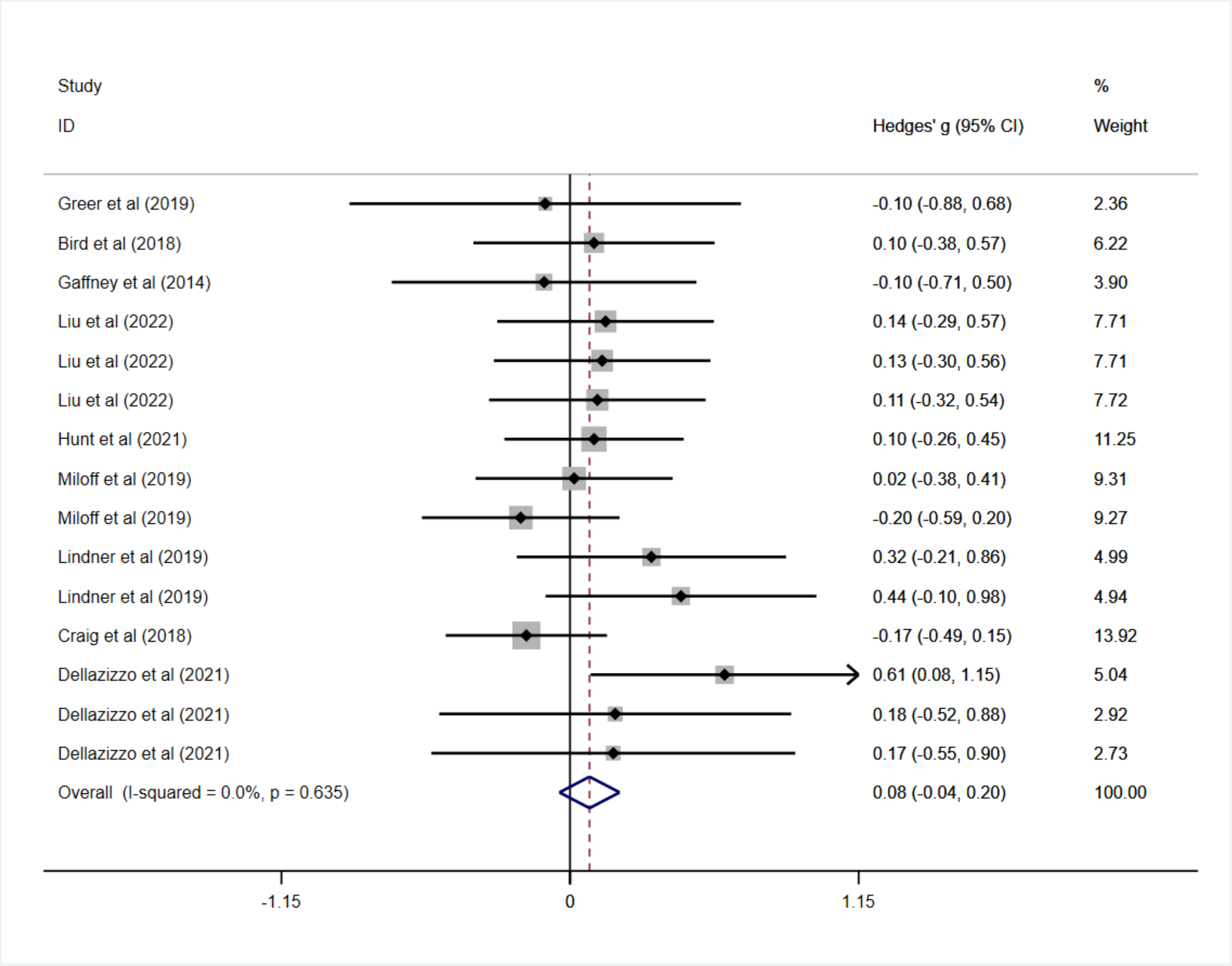

Figure S8. Forest plot for the short-term effects of CAIs on specific anxiety symptoms

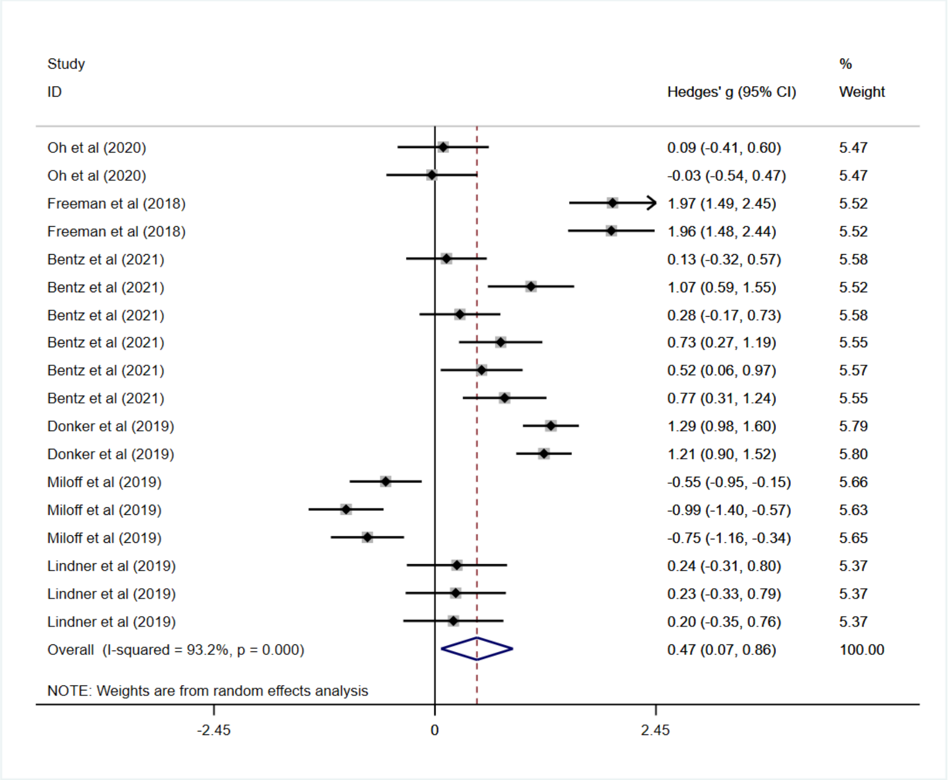

Figure S9. Forest plot for the long-term effects of CAIs on specific anxiety symptoms

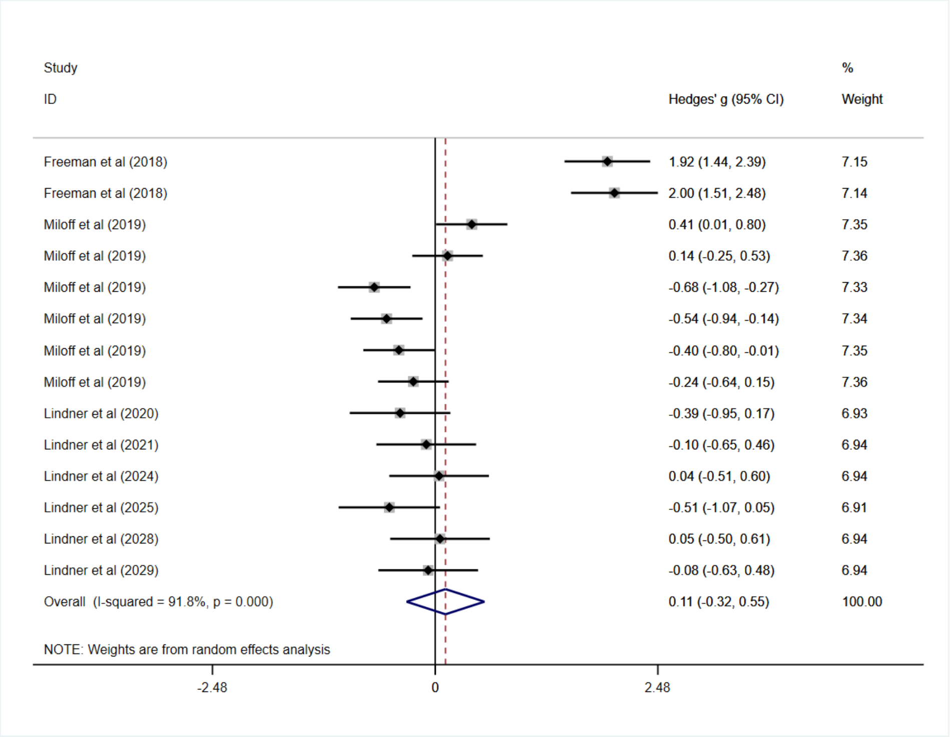

Figure S10. Subgroup analyses of efficacy of CAIs for specific anxiety symptoms

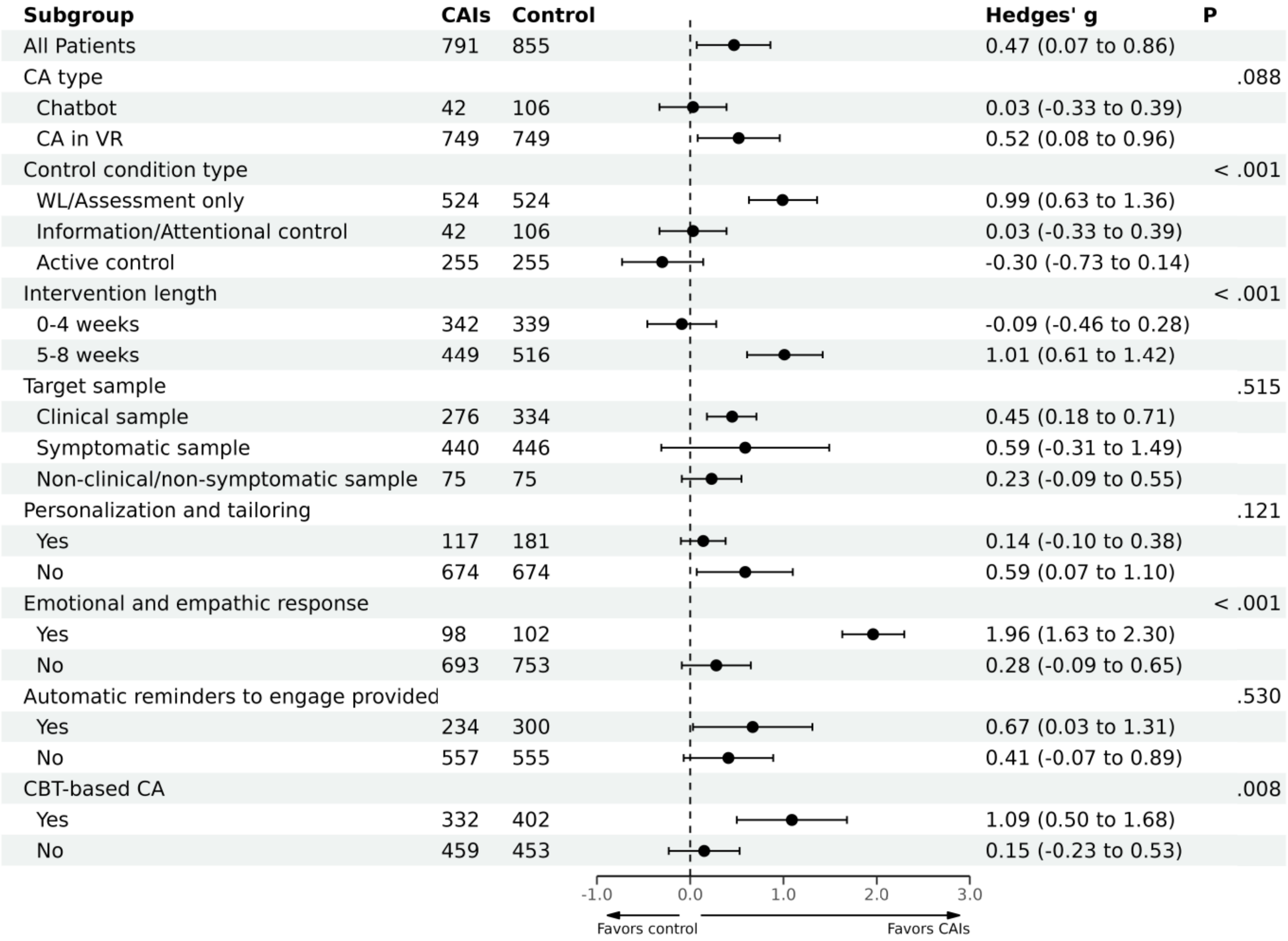

P value represents the significance of the Q-test. CA, conversational agent; ECA, embodied conversational agent; VR, virtual reality; CBT, cognitive behavioral therapy.

Figure S11. Forest plot for the short-term effects of CAIs on quality of life

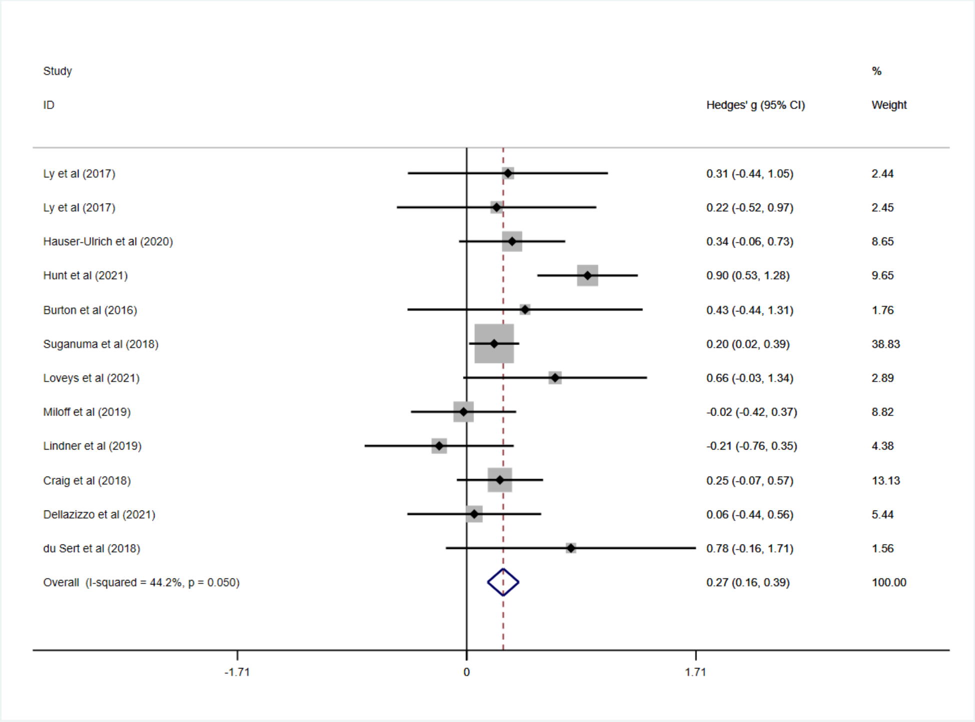

Figure S12. Forest plot for the long-term effects of CAIs on quality of life

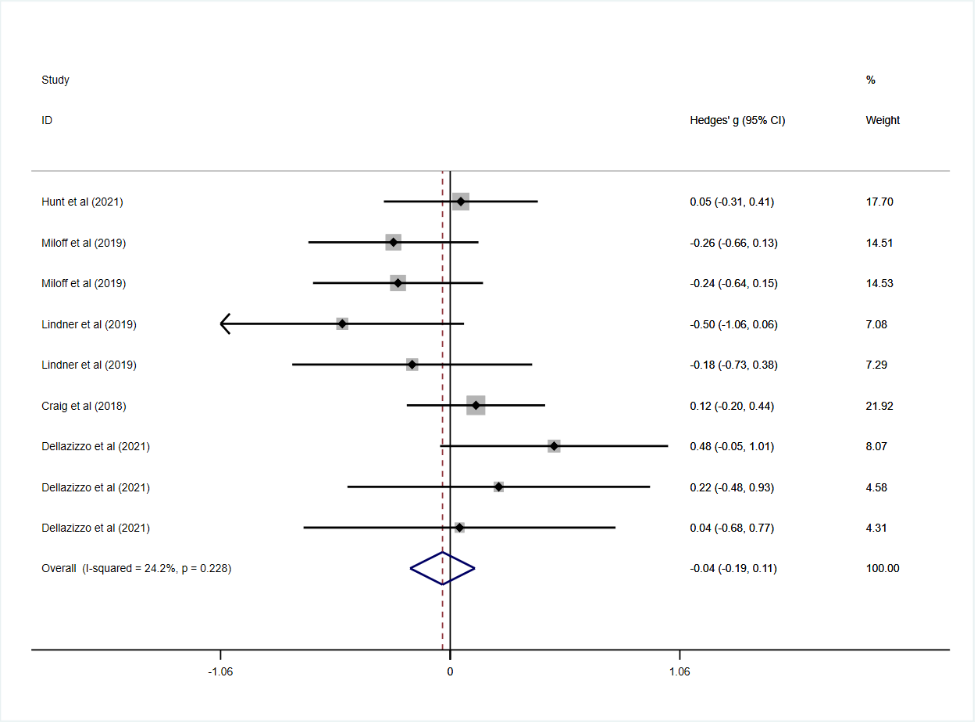

Figure S13. Subgroup analyses of efficacy of CAIs for quality of life

Subgroup	CAIs	Control	Hedges' g	P
All Patients	561	607	0.27 (0.16 to 0.39)	
CA type				.041
Chatbot	149	130	0.54 (0.26 to 0.83)	
ECA	218	287	0.21 (0.03 to 0.40)	
CA in VR	75	75	-0.08 (-0.40 to 0.24)	
Avatar	119	115	0.24 (-0.02 to 0.50)	
Control condition type				.072
WL/Assessment only	355	408	0.44 (0.16 to 0.71)	
Active control	206	199	0.13 (-0.07 to 0.32)	
Intervention target				.486
Directly aimed at this outcome	219	291	0.21 (0.04 to 0.39)	
Not directly aimed at this outcome	342	316	0.32 (0.07 to 0.58)	
Intervention length				.355
0-4 weeks	118	118	0.10 (-0.16 to 0.39)	
5-8 weeks	12	9	0.43 (-0.44 to 1.31)	
9+ weeks	431	480	0.37 (0.13 to 0.62)	
Target sample				.342
Clinical sample	116	117	0.21 (-0.05 to 0.47)	
Symptomatic sample	201	174	0.49 (0.10 to 0.88)	
Non-clinical/non-symptomatic sample	244	316	0.17 (0.01 to 0.34)	
Personalization and tailoring				.246
Yes	172	168	0.18 (-0.04 to 0.39)	
No	389	439	0.39 (0.10 to 0.67)	
Emotional and empathic response				.047
Yes	233	210	0.43 (0.20 to 0.66)	
No	328	397	0.14 (-0.03 to 0.30)	
Automatic reminders to engage provided				.408
Yes	86	67	0.42 (0.10 to 0.74)	
No	475	540	0.26 (0.04 to 0.48)	
CBT-based CA				.075
Yes	367	409	0.43 (0.18 to 0.69)	
No	194	198	0.12 (-0.10 to 0.35)	

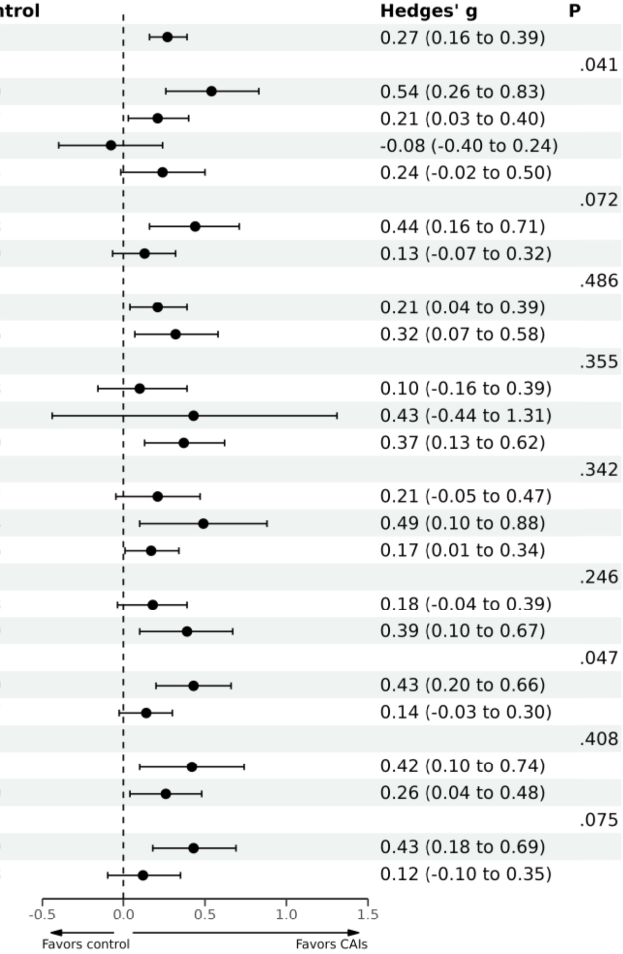

P value represents the significance of the Q-test. CA, conversational agent; ECA, embodied conversational agent; VR, virtual reality; CBT, cognitive behavioral therapy.

Figure S14. Forest plot for the short-term effects of CAIs on general distress

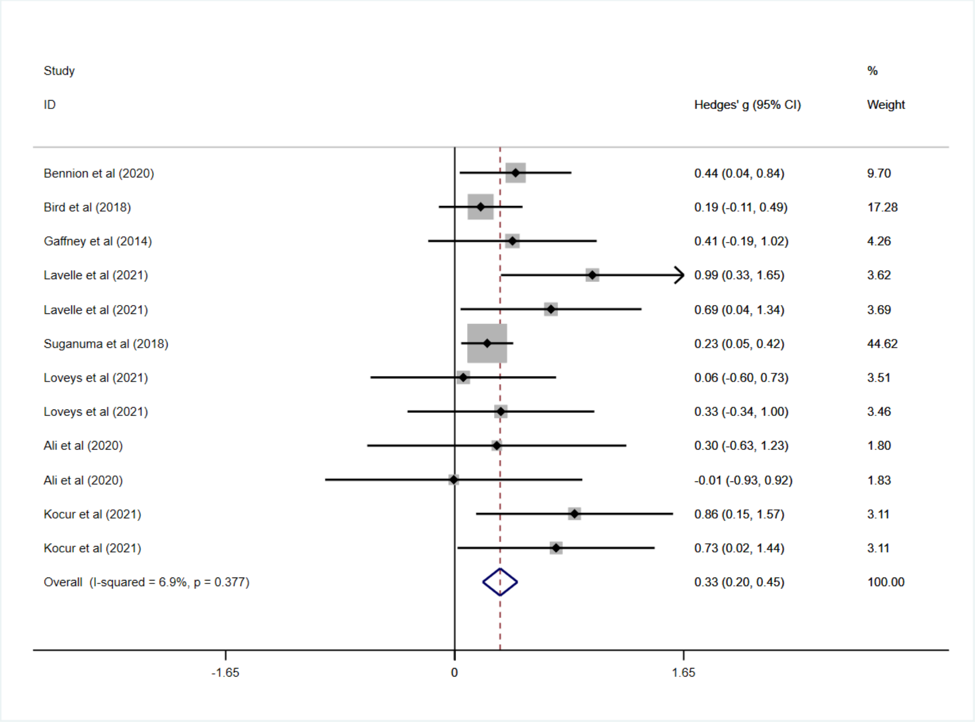

Figure S15. Forest plot for the long-term effects of CAIs on general distress

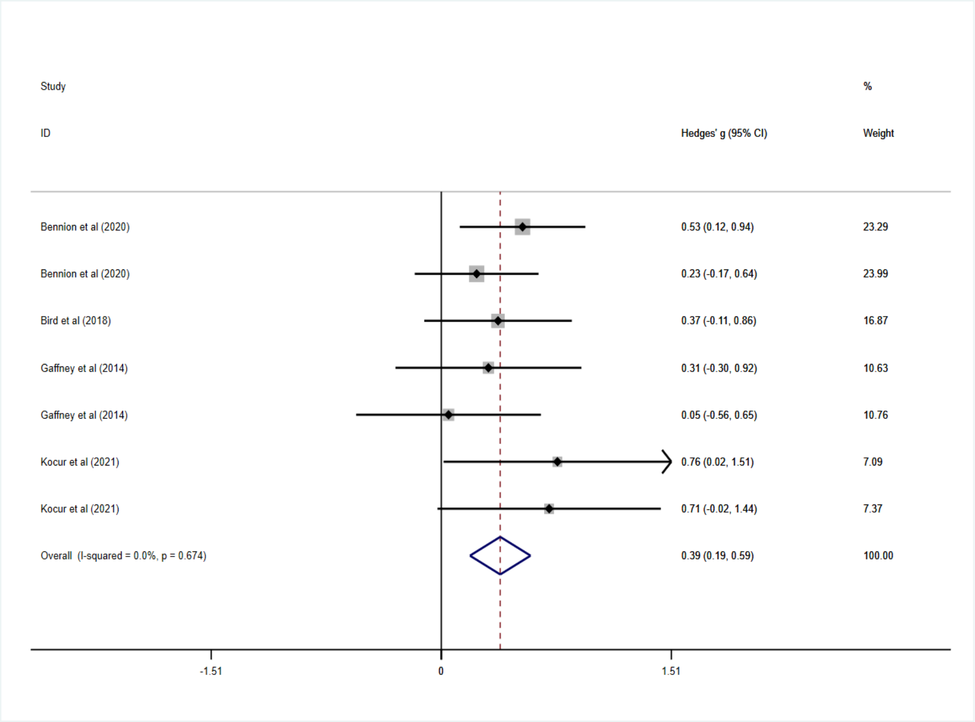

Figure S16. Subgroup analyses of efficacy of CAIs for general distress

Subgroup	CAIs	Control	Hedges' g	P
All Patients	473	536	0.33 (0.20 to 0.45)	
CA type				.059
Chatbot	199	193	0.45 (0.19 to 0.71)	
ECA	239	311	0.22 (0.06 to 0.39)	
Avatar	35	32	0.80 (0.29 to 1.30)	
Control condition type				.772
WL/Assessment only	265	329	0.40 (0.11 to 0.69)	
Information/Attentional control	18	18	0.15 (-0.51 to 0.80)	
Active control	190	189	0.39 (0.17 to 0.61)	
Intervention target				.110
Directly aimed at this outcome	267	258	0.43 (0.25 to 0.60)	
Not directly aimed at this outcome	206	278	0.22 (0.04 to 0.40)	
Intervention length				.110
0-4 weeks	264	255	0.44 (0.25 to 0.64)	
9+ weeks	209	281	0.23 (0.05 to 0.41)	
Target sample				.612
Clinical sample	53	50	0.56 (0.16 to 0.95)	
Symptomatic sample	137	137	0.22 (-0.01 to 0.46)	
Non-clinical/non-symptomatic sample	283	349	0.48 (0.17 to 0.78)	
Personalization and tailoring				.002
Yes	79	68	0.82 (0.48 to 1.16)	
No	394	468	0.25 (0.12 to 0.38)	
Emotional and empathic response				.028
Yes	109	98	0.61 (0.33 to 0.89)	
No	364	438	0.25 (0.11 to 0.39)	
Automatic reminders to engage provided				.096
Yes	203	205	0.27 (0.07 to 0.46)	
No	270	331	0.62 (0.25 to 0.98)	
CBT-based CA				.836
Yes	244	313	0.35 (0.10 to 0.60)	
No	229	223	0.38 (0.18 to 0.58)	

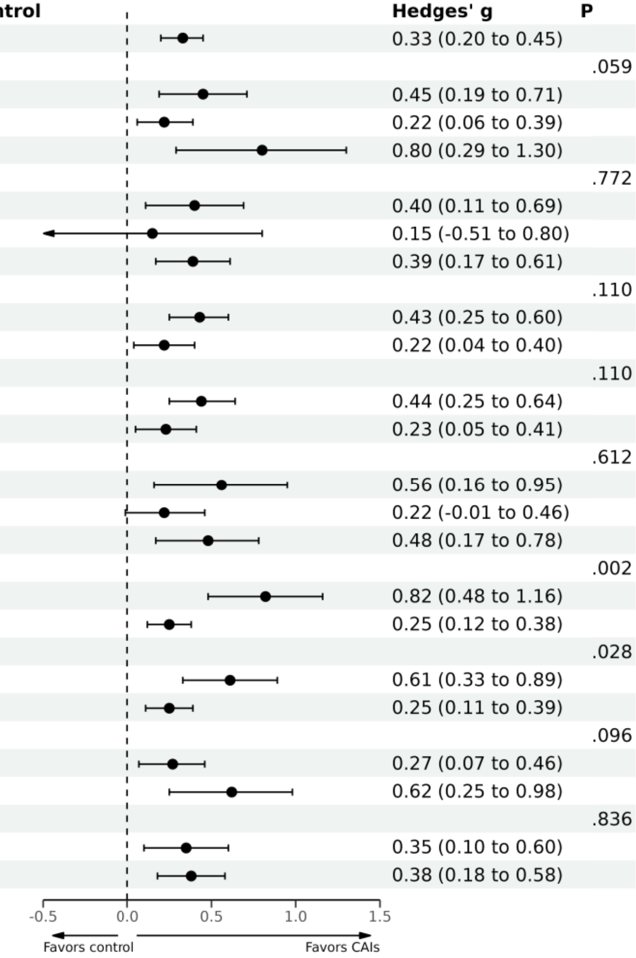

P value represents the significance of the Q-test. CA, conversational agent; ECA, embodied conversational agent; VR, virtual reality; CBT, cognitive behavioral therapy.

Figure S17. Forest plot for the short-term effects of CAIs on stress

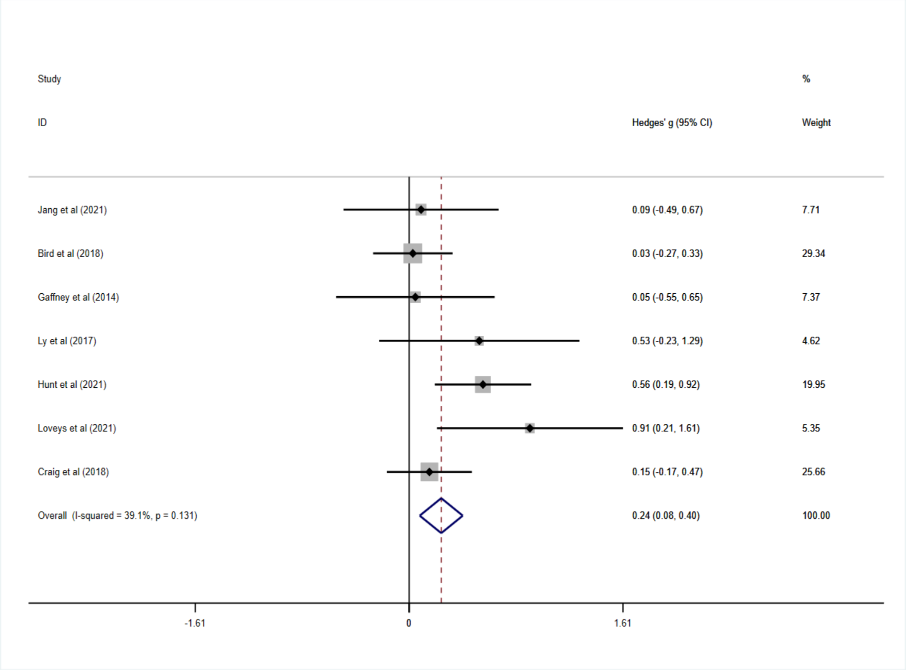

Figure S18. Forest plot for the long-term effects of CAIs on stress

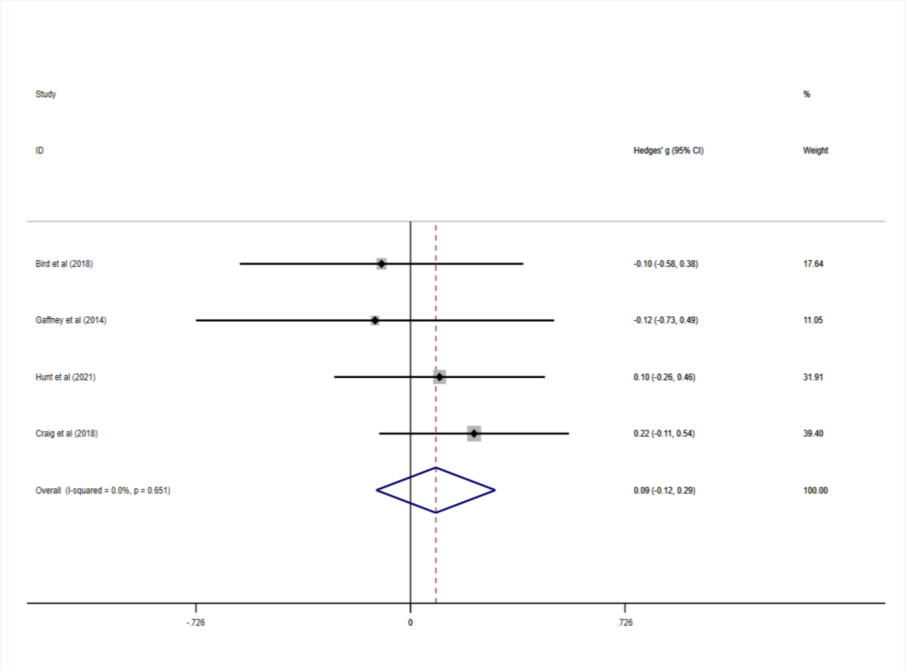

Figure S19. Subgroup analyses of efficacy of CAIs for stress

Subgroup	CAIs	Control	Hedges' g	P
All Patients	296	292	0.24 (0.08 to 0.41)	
CA type				.152
Chatbot	206	202	0.24 (-0.02 to 0.49)	
ECA	15	15	0.91 (0.21 to 1.61)	
Avatar	75	75	0.15 (-0.17 to 0.47)	
Control condition type				.013
WL/Assessment only	91	88	0.62 (0.32 to 0.91)	
Information/Attentional control	23	22	0.09 (-0.50 to 0.68)	
Active control	182	182	0.08 (-0.12 to 0.29)	
Intervention target				.054
Directly aimed at this outcome	29	29	0.73 (0.22 to 1.25)	
Not directly aimed at this outcome	267	263	0.19 (-0.02 to 0.40)	
Intervention length				.772
0-4 weeks	136	136	0.30 (-0.10 to 0.70)	
5-8 weeks	23	22	0.09 (-0.49 to 0.67)	
9+ weeks	137	134	0.35 (-0.05 to 0.74)	
Target sample				.514
Clinical sample	98	97	0.14 (-0.14 to 0.42)	
Symptomatic sample	184	181	0.34 (-0.04 to 0.73)	
Non-clinical/non-symptomatic sample	14	14	0.53 (-0.23 to 1.29)	
Personalization and tailoring				.707
Yes	89	89	0.21 (-0.09 to 0.51)	
No	207	203	0.29 (-0.02 to 0.61)	
Emotional and empathic response				.042
Yes	166	163	0.46 (0.14 to 0.77)	
No	130	129	0.04 (-0.20 to 0.29)	
Automatic reminders to engage provided				.463
Yes	145	144	0.20 (-0.15 to 0.54)	
No	151	148	0.37 (0.07 to 0.66)	
CBT-based CA				.094
Yes	99	95	0.44 (0.16 to 0.73)	
No	197	197	0.13 (-0.10 to 0.36)	

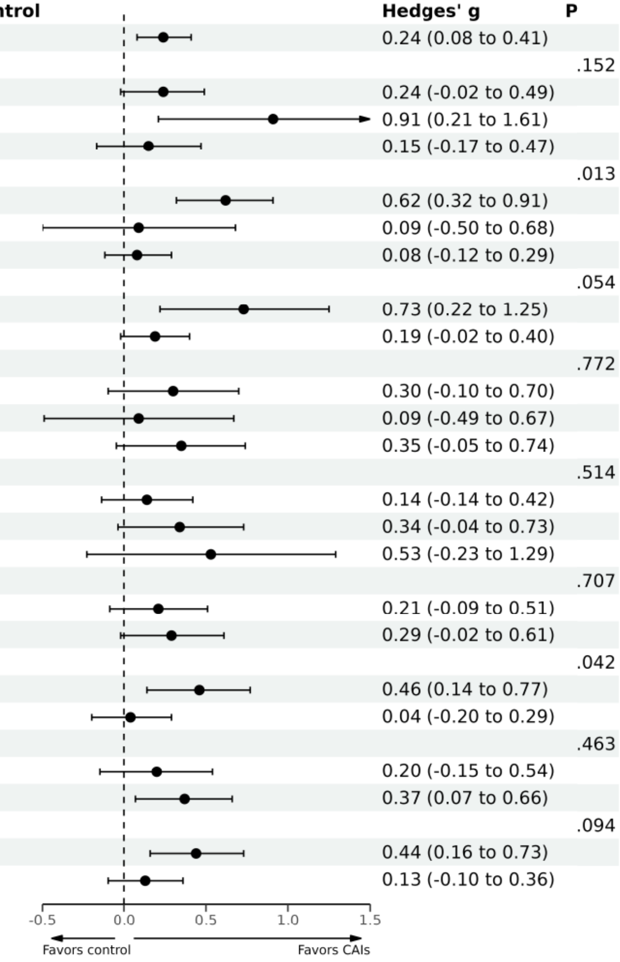

P value represents the significance of the Q-test. CA, conversational agent; ECA, embodied conversational agent; VR, virtual reality; CBT, cognitive behavioral therapy.

Figure S20. Forest plot for the short-term effects of CAIs on other outcomes

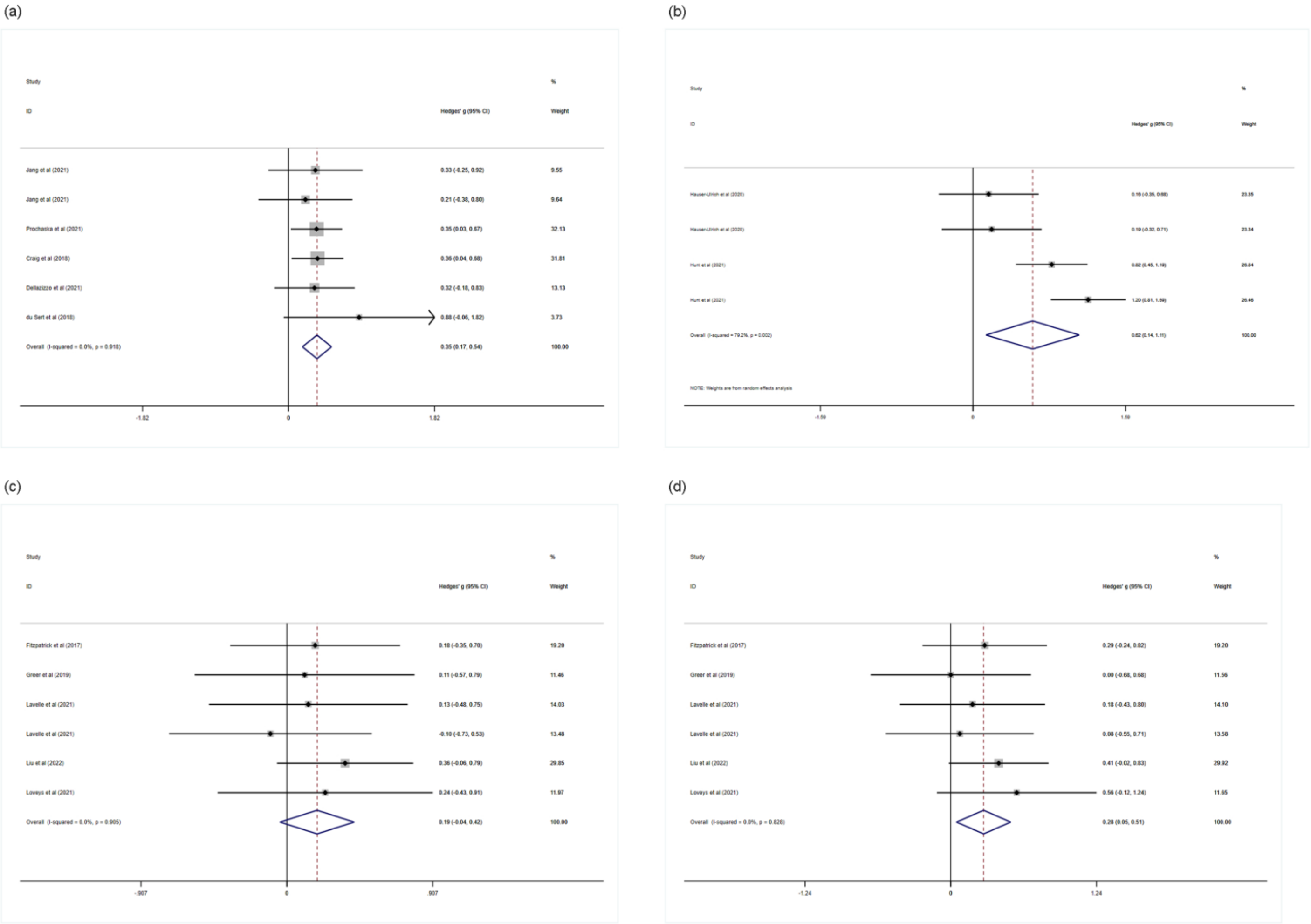

(a) effects on mental disorder symptoms. **(b)** effects on psychosomatic disease symptoms. **(c)** effects on positive affect. **(d)** effects on negative affect.

Figure S21. Forest plot for the long-term effects of CAIs on other outcomes

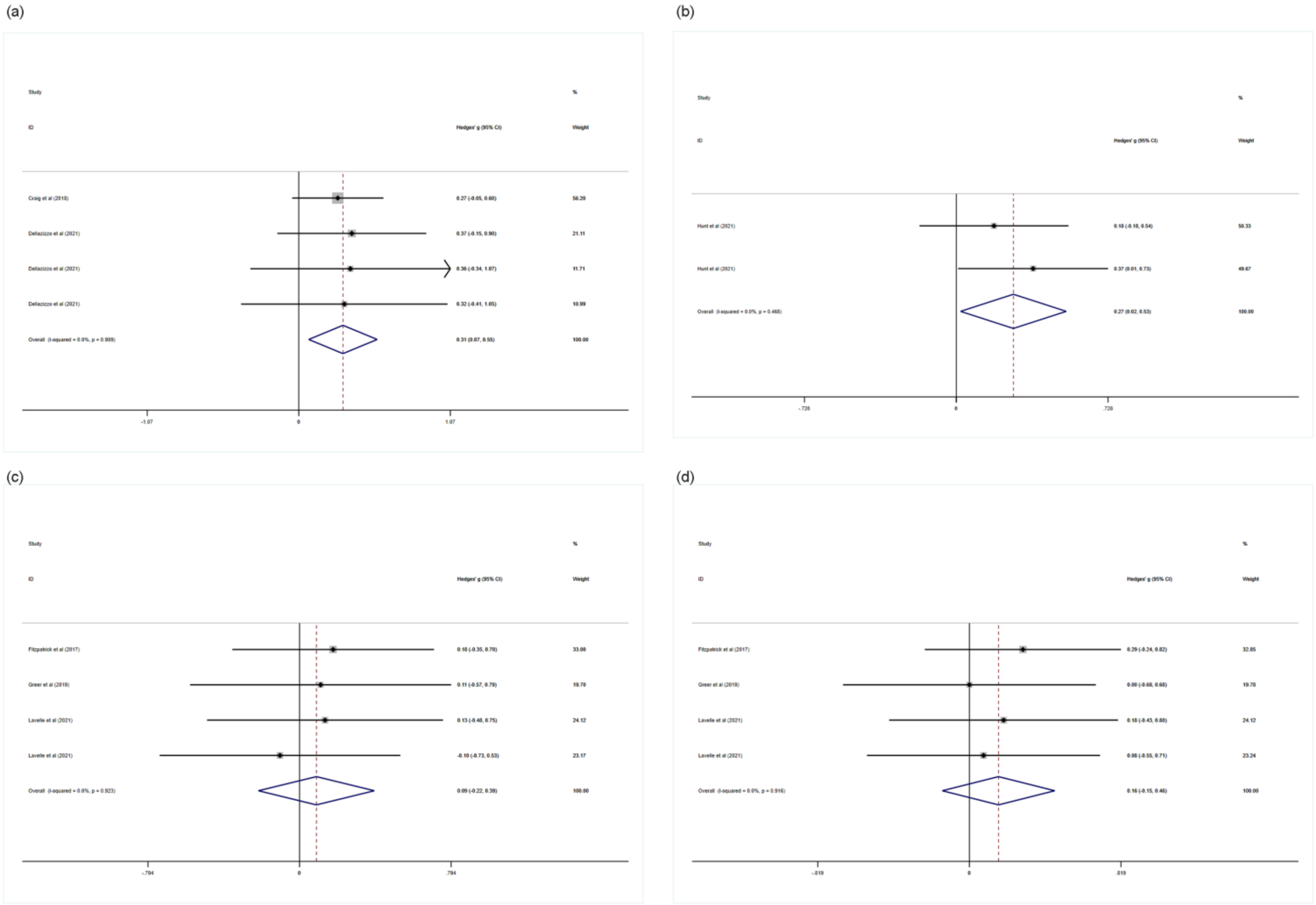

(a) effects on mental disorder symptoms. **(b)** effects on psychosomatic disease symptoms. **(c)** effects on positive affect. **(d)** effects on negative affect.
